# Integration of Within-Cell Experimental Data With Multi-Compartmental Modeling Predicts H-Channel Densities and Distributions in Hippocampal OLM Cells

**DOI:** 10.3389/fncel.2020.00277

**Published:** 2020-09-17

**Authors:** Vladislav Sekulić, Feng Yi, Tavita Garrett, Alexandre Guet-McCreight, J. Josh Lawrence, Frances K. Skinner

**Affiliations:** ^1^Krembil Research Institute, University Health Network, Toronto, ON, Canada; ^2^Department of Physiology, University of Toronto, Toronto, ON, Canada; ^3^Department of Biomedical and Pharmaceutical Sciences, Center for Biomolecular Structure and Dynamics, Center for Structural and Functional Neuroscience, University of Montana, Missoula, MT, United States; ^4^Neuroscience Graduate Program and Vollum Institute, Oregon Health & Science University, Portland, OR, United States; ^5^Department of Pharmacology and Neuroscience, Texas Tech University Health Sciences Center, Lubbock, TX, United States; ^6^Center for Translational Neuroscience and Therapeutics, Texas Tech University Health Sciences Center, Lubbock, TX, United States; ^7^Garrison Institute on Aging, Texas Tech University Health Sciences Center, Lubbock, TX, United States; ^8^Departments of Medicine (Neurology) and Physiology, University of Toronto, Toronto, ON, Canada

**Keywords:** hippocampus, interneuron, inhibitory cell, dendrite, h-channels, multi-compartment model, electrophysiology

## Abstract

Determining biophysical details of spatially extended neurons is a challenge that needs to be overcome if we are to understand the dynamics of brain function from cellular perspectives. Moreover, we now know that we should not average across recordings from many cells of a given cell type to obtain quantitative measures such as conductance since measures can vary multiple-fold for a given cell type. In this work we examine whether a tight combination of experimental and computational work can address this challenge. The oriens-lacunosum/moleculare (OLM) interneuron operates as a “gate” that controls incoming sensory and ongoing contextual information in the CA1 of the hippocampus, making it essential to understand how its biophysical properties contribute to memory function. OLM cells fire phase-locked to the prominent hippocampal theta rhythms, and we previously used computational models to show that OLM cells exhibit high or low theta spiking resonance frequencies that depend respectively on whether their dendrites have hyperpolarization-activated cation channels (h-channels) or not. However, whether OLM cells actually possess dendritic h-channels is unknown at present. We performed a set of whole-cell recordings of OLM cells from mouse hippocampus and constructed three multi-compartment models using morphological and electrophysiological parameters extracted from the same OLM cell, including per-cell pharmacologically isolated h-channel currents. We found that the models best matched experiments when h-channels were present in the dendrites of each of the three model cells created. This strongly suggests that h-channels must be present in OLM cell dendrites and are not localized to their somata. Importantly, this work shows that a tight integration of model and experiment can help tackle the challenge of characterizing biophysical details and distributions in spatially extended neurons. Full spiking models were built for two of the OLM cells, matching their current clamp cell-specific electrophysiological recordings. Overall, our work presents a technical advancement in modeling OLM cells. Our models are available to the community to use to gain insight into cellular dynamics underlying hippocampal function.

## Introduction

The challenge of understanding brain function given its many cell types and circuits is being greatly aided by the development of sophisticated experimental techniques, big data, and interdisciplinary collaborations (Ecker et al., 2017). Furthermore, the use of computational brain models is becoming established as an important tool that can bridge across scales and levels (Cutsuridis et al., 2010; O'Leary et al., 2015; Bassett et al., 2018). It is now clear that it is essential to consider the unique contributions of specific cell types and circuits in order to understand brain behavior (Luo et al., 2018). In particular, we know that different inhibitory cell types can control circuit output and brain function in specific ways (Kepecs and Fishell, 2014; Roux and Buzsáki, 2015; Abbas et al., 2018; Cardin, 2018) and, by extension, disease states (Marín, 2012; Giovannetti and Fuhrmann, 2019).

The contribution of a specific cell type to network and behavioral function is necessarily grounded in its biophysical properties. While immunohistochemical and single-cell transcriptomic studies provide insight into which ion channels might be present in a particular cell type, how different cell types contribute to function must necessarily include their activity within circuits (Kopell et al., 2014). An individual neuron's electrical activity largely arises from its ion channel kinetics, densities, and localization across its neuronal compartments. In this regard, mathematical multi-scale (channel and cellular), multi-compartment computational models are needed to help provide insights and hypotheses of how specific cell types contribute to brain function and disease processes. However, creating such models requires quantitative knowledge of the precise characteristics of the particular cell type, and it is highly challenging, if not impossible, to obtain comprehensive knowledge of all the relevant biophysical parameters of each compartment of each cell type experimentally. All experiments come with their own set of caveats and limitations, and mathematical models, no matter how detailed, are always a simplification relative to the biology. It is therefore important not to lose sight of the limitations of both model and experiment by having an ongoing dialogue between the two.

It is now well-known that the characteristics of a given cell type are not fixed (Marder and Goaillard, 2006), and thus a component of experimental variability reflects heterogeneity inherent in specific neuronal populations and thus in circuits. Moreover, such variability is likely to be functionally important (Wilson, 2010). Previous work has shown that conductance densities for a given ion channel in an identified cell type can have a two- to six-fold range of values (Goaillard et al., 2009; Ransdell et al., 2013). Despite this variability in channel conductances, robust neuronal as well as circuit output is maintained, as most clearly shown in the crustacean stomatogastric ganglion circuit (Bucher et al., 2005; Schulz et al., 2006; Tang et al., 2012). The conservation of individual neuronal electrical output despite variable underlying ion channel conductance densities has furthermore been demonstrated in mammalian CNS neurons (Swensen and Bean, 2005), most likely arising from complex homeostatic mechanisms for maintaining circuit stability that are not fully understood. How should one proceed in building cellular, computational models? Averaging of experimental variables such as conductance densities as a way of accounting for variability leads to erroneous conductance-based models (Golowasch et al., 2002). As a consequence, single, “canonical” biophysical models cannot capture inherent variability in the experimental ion channel data. A more desirable approach is to develop multiple models to capture the underlying biological variability (Marder and Taylor, 2011). Indeed, such populations of models representing a given cell type have been developed to examine, for example, co-regulations between different conductances that might exist in a given cell type (Hay et al., 2011; Soofi et al., 2012; Sekulić et al., 2014). In this way, populations of models could help suggest what balance of conductances are important for cellular dynamics and their function in circuits. Ideally, one should obtain biophysical properties of a given cell type using recordings from the *same* cell. It is of course unrealistic to consider an experimental characterization of all the various ion channel types using the same cell of a given cell type. This impracticality is further enhanced in consideration of channel types in the dendrites of neurons. Besides needing to patch from the same cell, there are also the practical limitations of invasively investigating the biophysical characteristics of fine dendritic compartments, performing multiple solution changes to pharmacologically isolate specific conductances, and acquiring high quality data within the time frame of optimal cell health. However, dendrites are where most synaptic contacts are made and where signal integration in neurons occurs (Stuart and Spruston, 2015). Thus, these aspects must be tackled along with considerations of cellular variability.

In this work we focus on the oriens-lacunosum/moleculare (OLM) cell, an identified inhibitory cell type in the hippocampal CA1 area (Maccaferri and Lacaille, 2003; Müller and Remy, 2014). OLM cells receive excitatory glutamatergic input predominantly from local CA1 pyramidal neurons and form GABAergic synapses onto the distal dendrites of CA1 pyramidal neurons, as well as onto other CA1 inhibitory cells (Blasco-Ibáñez and Freund, 1995; Maccaferri et al., 2000; Klausberger, 2009; Leão et al., 2012). Functionally, proposed roles of OLM cells include gating sensory and contextual information in CA1 (Leão et al., 2012), and supporting the acquisition of fear memories (Lovett-Barron et al., 2014). Moreover, OLM cell firing is phase-locked to the prominent theta rhythms in the hippocampus of behaving animals (Klausberger et al., 2003; Klausberger and Somogyi, 2008; Varga et al., 2012; Katona et al., 2014). Although it has long been known that OLM cells express hyperpolarization-activated cation channels (h-channels) (Maccaferri and McBain, 1996), it is still unclear whether these channels are present in their dendrites. From a functional perspective, the consequences of dendritic h-channel expression in OLM cells was explored in our previous computational study where h-channels were found to modulate the spiking preference of OLM cell models—incoming inhibitory inputs recruited either a higher or lower theta frequency (akin to Type 1 or Type 2 theta, respectively—Kramis et al., 1975) depending on the presence or absence of dendritic h-channels (Sekulić and Skinner, 2017). In that computational study, our OLM cell models were derived from previously built populations of OLM cell multi-compartment models in which appropriate OLM cell models were found with h-channels present either in the soma only or uniformly distributed in the soma and dendrites (Sekulić et al., 2014). We had previously leveraged these models and showed that appropriate OLM cell model output could be maintained, even if h-channel conductance densities and distributions co-vary, so long as total membrane conductance due to h-channels is conserved (Sekulić et al., 2015)—a finding that was also demonstrated in cerebellar Purkinje neurons (Angelo et al., 2007). Moreover, these OLM cell models were developed using morphological and electrophysiological data obtained from different OLM cells as well as h-channel characteristics from the literature, resulting in non-uniqueness of the fitted model parameters (Rall et al., 1992; Holmes et al., 2006). However, we do not actually know whether h-channels are present in the dendrites of OLM cells. The existence of dendritic h-channels has not been directly assessed using patch-clamp recordings from OLM cell dendrites, and immunohistochemistry studies have so far demonstrated expression of the HCN2 subunit of h-channels only in the somata of OLM cells (Matt et al., 2011; Hilscher et al., 2019).

Considering all of the above, in this paper we aimed to build “next generation” multi-compartment models of OLM cells to achieve a two-pronged goal. First, to determine whether multi-compartment models built using morphological and electrophysiological data from the *same* cell would produce *consistent* results regarding h-channel localization to dendrites or not, and second, to determine the biophysical characteristics of h-channels in OLM cells. We consider the models developed here to be next generation relative to previous multi-compartment OLM cell modeling efforts (Lawrence et al., 2006; Sekulić et al., 2014) because each model was built using experimental data from the same cell, including its morphology, passive properties, and biophysical h-channel characteristics. Using transgenic mice in which yellow fluorescent protein (YFP) was expressed in somatostatin (SOM)-containing neurons, we visually targeted OLM cells from CA1 hippocampus, and fully reconstructed three OLM cells for multi-compartment model development with h-channel characteristics fit to each particular cell. We found that in order to be compatible with the experimental data, all three models needed to have h-channels present in their dendrites. Using two of these reconstructed models, we completed their development into full spiking models by including additional ion channel currents whose parameters were optimized based on voltage recordings from the same cell. These resulting models and associated experimental data are publicly available and can be subsequently used to develop further insight into the biophysical specifics of OLM cells and to help understand their contributions to circuit dynamics and behavior. This work demonstrates the feasibility of combining experimental and computational studies to address the challenging issue of determining the density and distribution of specific dendritic ion channel types.

## Materials and Methods

### Ethics Statement

All procedures were performed in accordance with the University of Montana (Animal Use Protocols 026-11 and 017-14) and Texas Tech University Health Sciences Center (Animal Use Protocols 15025, 15031, and 16037) Institutional Animal Care and Use Committees.

### Animals and Brain Slice Preparation

Heterozygous crosses of homozygous somatostatin-CRE mice (SOM-CRE; stock no. 013044; Jackson Labs) and Rosa26YFP mice (Jackson Labs stock no. 007920) were obtained as previously described (Yi et al., 2014). Transverse hippocampal slices were prepared as described previously (Yi et al., 2014). Briefly, SOM-CRE^+/−^:Rosa26YFP^+/−^ (SOM-YFP) mice of both sexes (9–10 weeks, *n* = 6) were anesthetized with isoflurane and then transcardially perfused with ice-cold partial sucrose solution (PSS) containing (mM): 80 NaCl, 2.5 KCl, 24 NaHCO_3_, 0.5 CaCl_2_, 4 MgCl_2_, 1.25 NaH_2_PO_4_, 25 glucose, 75 sucrose, 1 ascorbic acid, 3 sodium pyruvate, saturated with 95% O2/5% CO_2_, pH 7.4 (Bischofberger et al., 2006). After carefully extracting, blocking, and mounting the brain, transverse hippocampal slices (300 μm) were cut in ice-cold oxygenated PSS with a 1200 S Vibratome (with Vibrocheck accessory; Leica Microsystems, Bannockburn, IL, USA), and then were incubated in warm (36°C) oxygenated PSS at least 30 min before use.

### Chemical Reagents

DL-APV was purchased from R&D Systems (Minneapolis, MN, USA). Tetrodotoxin (cat# 5651), TEA (cat# 2265), 4-AP (cat# A78403), DNQX (cat# D0540), SR-95531 (cat# S106), and ZD7288 hydrate (cat# Z3777) were purchased from Sigma-Aldrich, Inc. (Saint Louis, MO, USA). Salts and chemicals for saline solutions, including biocytin, were also purchased from Sigma-Aldrich, Inc.

### Electrophysiological Recordings and Analyses

Hippocampal slices were transferred to a recording chamber and submerged in artificial cerebrospinal fluid (ACSF) solution containing (mM): 125 NaCl, 2.5 KCl, 25 NaHCO_3_, 2 CaCl_2_, 1 MgCl_2_, 1.25 NaH_2_PO_4_ and 20 glucose, saturated with 95% O_2_/5% CO_2_, pH7.4, at 34–35°C. SOM-YFP cells in the CA1 stratum oriens layer of hippocampus were visualized using IR-Dodt contrast and fluorescence video-microscopy (Zeiss Axiovision 4.7) on either a Patch Pro 2000 (Scientifica Ltd, Uckfield, East Sussex, UK) or Infrapatch (Luigs and Neumann, Ratingen, Germany) on an upright Zeiss microscope (Axio Examiner; Carl Zeiss Microscopy, LLC, Thornwood, NY, USA). On the Patch Pro 2000, live YFP-positive cells were visualized with a 505 nm LED (LED4C11-SP; Thorlabs) driven by a four-channel LED driver (DC4100; Thorlabs). On the Infrapatch rig, a 505 nm LED was controlled by the Colibri LED illumination system (Carl Zeiss Microscopy). Patch pipettes (2–4 MΩ) were fabricated using a two-step vertical electrode puller (PC-10; Narishige, East Meadow, NY, USA) and filled with internal solution containing (mM): 110 potassium gluconate, 40 KCl, 10 HEPES, 0.1 EGTA, 4 MgATP, 0.3 Na_2_GTP, 10 Na_2_ phosphocreatine and biocytin 0.2%, titrated to pH 7.2 with KOH, osmolarity 295–305 mOsm/L. Whole cell recordings were made using a Multiclamp 700B amplifier (Molecular Devices, Union City, CA, USA), filtered at 4 kHz, and digitized at 20 kHz (Digidata 1440A; Molecular Devices). Current and voltage traces were acquired on a PC running Axograph X (Axograph Scientific, Sydney, Australia). Solutions were heated to 34–35°C with an inline solution heater (HPT-2, Scientifica; SH-27B/TC-324B, Warner, Hamden, CT, USA). Access resistance (R_s_) was monitored during recording. Cells with initial R_s_ less than 20 MΩ were recorded. If R_s_ changed more than 20% during the course of the whole cell recording, the data were excluded from further analyses. In all recordings, the AMPA receptor antagonist DNQX (25 μM), the NMDA receptor antagonist DL-APV (50 μM), and the GABA_A_ receptor antagonist SR-95531 (gabazine; 5 μM) were included in the ACSF. For blocking intrinsic voltage-gated channels to isolate h-channel currents (*I*_*h*_), TEA (10 mM), 4-AP (5 mM), and TTX (1 μM) were applied. The h-channel specific blocker ZD7288 (10 μM) was used to obtain and constrain *I*_*h*_ parameters on a per-cell basis.

The order of protocols is important to consider during the subsequent procedures of obtaining OLM cell passive properties in light of varying stages of cell health and deterioration as the recordings progressed. The chronological order of current clamp and voltage clamp experimental protocols performed are shown in Table 1. The approximate length of experiment for a given cell patched was at most 30 min. At the end of the recording, pipettes were withdrawn to outside-out patch configuration. Slices were kept on the rig for several minutes to facilitate diffusion of biocytin to distant subcellular compartments. Electrophysiological data were analyzed with Axograph X. The junction potential was calculated to be 11.88 mV and was subtracted from all experimentally recorded voltage values prior to use in subsequent data analysis and creation of multi-compartment computational models.

**Table 1:** Experimental protocols performed on OLM cells.

**Order**	**Bath solution**	**Recording mode**	**Description of protocol**
#1	ACSF	VC at −60 mV	Seal test
#2	ACSF + DNQX/APV/Gabazine	CC at −60 mV	2s-long steps from −120 to +90 pA
			in 30 pA steps
#3	ACSF + DNQX/APV/Gabazine	VC at −40 mV	*I*_*h*_ activation: 1.2 s-long step at
			progressively hyperpolarized potentials
			to −120 mV in −10 mV increments
#4	ACSF + DNQX/APV/Gabazine	CC at −60 mV	Same protocol as #2
	+ TTX/4-AP/TEA		
#5	ACSF + DNQX/APV/Gabazine	VC at −40 mV	Same protocol as #3
	+ TTX/4-AP/TEA		
#6	ACSF + DNQX/APV/Gabazine	VC at −40 mV	*I*_*h*_ tail currents: a prepulse to −120 mV
	+ TTX/4-AP/TEA		for 1.2 s to fully activate h-channels.
			Then, a depolarized relaxation step
			at −110 mV was performed for 1 s
			before returning to the holding potential.
			Repeated multiple times,
			with the relaxation steps becoming
			successively more depolarized at 10 mV
			intervals across each repeated sweep.
#7	ACSF + DNQX/APV/Gabazine	CC at −60 mV	Same protocol as #2
	+ TTX/4-AP/TEA + ZD		
#8	ACSF + DNQX/APV/Gabazine	VC at −40 mV	Same protocol as #3
	+ TTX/4-AP/TEA + ZD		

*The order column displays the sequential order in which the protocols were performed*.

*Reagents concentration: DNQX (25 μM), APV (50 μM), Gabazine (5 μM), TEA (10 mM), 4-AP (5 mM), TTX (1 μM), ZD (10 μM). Further details in section Methods. VC, voltage clamp; CC, current clamp*.

### Visualization of Biocytin-Filled Cells and Confocal Imaging

During electrophysiological experiments, recorded SOM-YFP cells were filled with biocytin for *post-hoc* morphological reconstruction. After recording, slices were fixed overnight at 4°C in 0.1 M phosphate-buffered saline (PBS) containing 4% paraformaldehyde. After several washes in PBS, and 2 h permeabilization with 0.3% Triton X-100 in PBS at room temperature, slices were incubated overnight at 16°C in PBS with Alexa 633-conjugated streptavidin (final concentration 1 μg/mL, catalogue no. S-21375; Invitrogen). Slices were cryopreserved in 30% sucrose containing PBS and then re-sectioned at 100–150 μm thickness using a sliding freezing microtome (HM430; Thermo Scientific, Waltham, MA, USA). After staining with Neurotrace 435/455 (1:100 in PBS) and mounting on gelatin-coated slides in Vectashield (catalogue no. H-1400; Vector Laboratories), sections were imaged with a Fluoview FV-1000 confocal imaging system (Olympus) with 4x, 25x, and 60x objectives. Tiled confocal stacks (800 × 800 pixels; 0.2 μm z-step) of SOM-YFP cells were flat projected, rotated and cropped in PhotoShop 13.0 or ImageJ for display.

### Morphological Reconstruction of OLM Cells

Confocal microscope images at 60X magnification were acquired for the cells used in this work. The field of view of each image was restricted to 200 × 200 μm, resulting in 2–11 image “stacks” per cell. Bitplane Imaris was used for viewing reconstructions in 3D and for validating the z-stack. The microscope step size was 0.2 μm in the Z-plane, resulting in 150–200 images per stack. Variation in contrast between stacks were likely due to photobleaching, as stacks acquired later in the image acquisition process for each cell were more apparently bleached than the ones acquired earlier. Bleach correction was performed using ImageJ (Schneider et al., 2012) by normalizing the contrast of all stacks for each cell according to the average intensity value across all stacks per cell. Stacks were then stitched together to recover the volume information for the entire cell. Stitching was performed using the XuvTools software package (Emmenlauer et al., 2009). We next performed volumetric reconstruction of the soma, dendrites, and axons. This was done using the freely-available Neuromantic software package that implements semi-automated tracing (Myatt et al., 2012).

### Criteria for Selection of OLM Cells

Patch clamp recordings were performed on a total of 45 cells from the stratum oriens of SOM-YFP cells from SOM-Cre/Rosa26YFP mice. After histological processing was complete, neurons were classified as OLM cells if they possessed a horizontally-oriented cell body and dendrites within the oriens layer and a major axon projecting perpendicularly with ramifications in the lacunosum/moleculare layer. Additional criteria were developed for stability of access and input resistance, completeness of electrophysiological protocols, and signal-to-noise level in both current and voltage clamp recordings. Only those cells that exhibited <20% change in input resistance over the course of the experiment were considered for further modeling. The full suite of electrophysiological protocols, including wash-in of ZD7288 blocker to be able to determine *I*_*h*_ was required to fulfill selection criteria. Of the 45 cells recorded from in total, 11 OLM cells met electrophysiological criteria for stability, completeness, and noise level. Of these 11 OLM cells, three (*Cell 1, Cell 2, Cell 3*) were advanced for subsequent detailed experimental analyses and multi-compartment computational model development. Over the course of the recordings for these three cells, the input resistances as determined from seal test recordings changed from: 260.5 to 216.9 *MΩ* (−16.7%) for *Cell 1*; 147.3 to 175.1 *MΩ* (+18.9%) for *Cell 2*; and 458.7 to 390.6 *MΩ* (−14.8%) for *Cell 3*. The sources of these modest changes in input resistance are not clear, but mechanical drift, activity-dependence (execution of many protocols), and intracellular dialysis are suspected to be contributing factors.

### Multi-Compartment Model Creation and Passive Property Fitting Considerations

Reconstructed OLM cells yielded soma and somatodendritic surface areas (μm^2^) of: 7,651 and 29,378 for *Cell 1*; 13,035 and 35,159 for *Cell 2*; 6,911 and 21,990 for *Cell 3*. The NEURON simulation environment (Hines and Carnevale, 2001) was used to create the multi-compartment models. Compartmentalization of the models was done using the *d*_λ_ rule where compartment lengths are set to a fraction of the length constant λ_*f*_, where *f*=100 Hz. We set the fraction of *d*_λ_ to be 0.1 for all models. The finalized number of compartments (after staggered re-fitting) in each of the model cells is: 303, 632, and 837 for *Cell 1, Cell 2*, and *Cell 3*, respectively.

We selected long current clamp steps for the fitting of passive membrane properties rather than shorter voltage clamp “seal test” protocols due to the incomplete clamping of the membrane by short voltage clamp steps (Holmes, 2010). Recordings with synaptic blockers in addition to potassium and sodium blockers are referred to as “*TTX traces”* (i.e., step #4 in Table 1), due to TTX application. Recordings with synaptic blockers obtained in the presence of h-channel blocker ZD7288 in addition to TTX/4-AP/TEA are referred to as “*ZD traces”* and are given by step #7 in Table 1. Recordings performed with synaptic- and all voltage-gated channels blocked (i.e., ZD traces) were initially considered preferable for passive membrane property fitting in the models. However, due to the possibility of changes in membrane responses as a function of the length of the recording session, we compared the membrane time constants (τ_*m*_) during the charging portion of the current clamp step for the voltage traces obtained across recordings with synaptic- and voltage-gated blockers applied. We found that the −30 pA ZD traces, being the last traces recorded in the session, showed noisier membrane responses compared to the −120 pA ZD traces obtained earlier. This manifested as an “undershoot” of the −30 pA ZD traces (see Supplementary Figure 1). After normalization of the traces was done, it was clear that the −30 pA ZD traces showed a marked slowing of τ_*m*_ compared to both the −120 pA ZD as well as −30 pA TTX traces, the latter two being largely overlapping. This demonstrated that in the case of the −30 pA TTX current injection, few or no h-channels were activated as the *V*_*m*_ response was nearly identical to that of the ZD traces.

We fitted the passive membrane properties of multi-compartment models, as well as the h-channel parameters, using a virtual current clamp and the Multiple Run Fitter (MRF) of the NEURON simulation environment (Hines and Carnevale, 2001). The −120 pA ZD traces (or TTX traces for fitting h-channels) for each cell were used as the experimental recording for which the models' *V*_*m*_ trajectories needed to match in response to −120 pA virtual current. During use of the MRF in NEURON for the passive property fitting procedure, certain regions of the traces were discounted from fitting, such as the first 500 ms portion so that initial model transients did not affect the fitting. Furthermore, because the charging portion of *V*_*m*_ was very short—on the order of 100 ms—it was given a greater weight value (10X) compared to the rest of the trace, in the MRF. For fitting passive responses, typically only the initial portion of the steady-state response during the hyperpolarizing current clamp step was used for fitting, that is, about 50–60 ms after the charging portion was completed. This was because for some cells, a small depolarization was present even under ZD7288 block, which could have been due to noise or the presence of another, unidentified inward current that was not blocked. For cells where no such depolarization was present, the entire current membrane trace during the current clamp step was used for fitting. Additionally, approximately 1 second of the trace after the current clamp step was completed was used for fitting for each cell, to ensure the model's response to the release from the current clamp was captured. When fitting with h-channels, the entire steady-state response was used for fitting the model parameters, but with 10X higher weights assigned to the charging portion of *V*_*m*_ portions of the trace, as when doing the passive fitting. Also, when fitting the h-channel parameters in the model using the TTX traces, the post-inhibitory rebound portion of the experimental traces were also considered during the fits. An exception was for *Cell 3* where the post-inhibitory rebound region was not included, since for this cell model, no appropriate fits of h-channel parameters that could also adequately capture the sag response could be found (see section Discussion).

In summary, the duration of the experimental recordings is from 0 to at least 5,000 ms, with the current clamp step starting after one second for two seconds, i.e., from 1,000 to 3,000 ms. Model traces from 500 to 5,000 ms were used in the MRF for fitting both passive and *I*_*h*_ parameters.

### Passive Membrane Model and Experiment Comparisons

Input resistance (*R*_*in*_) in the passive models computed using a current clamp protocol of −120 pA, i.e., the same protocol used to fit the passive properties, is given by values of *V*_*m*_ taken at the start and end of the current clamp step: *R*_*in*_=(*V*_*start*_ − *V*_*end*_)/(120*pA*). Using experimental −120 pA ZD traces, the input resistance is also computed. These computed values are: *R*_*in*_ (MΩ) (passive model) = 411 (*Cell 1*), 332 (*Cell 2*), and 550 (*Cell 3*); *R*_*in*_ (MΩ) (expt with ZD7288) = 363 (*Cell 1*), 326 (*Cell 2*), and 531 (*Cell 3*).

We note that for the comparison of membrane time constants (τ_*m*_) of the OLM cells used to the models, we fitted exponential curves to the charging portion of *V*_*m*_ for each cell at various time points of the recording session using a nonlinear least squares regression (see above). The amplitude of the traces were normalized at the time point at which depolarizing responses in the TTX traces due to *I*_*h*_ cause the membrane potential to deviate from the (putatively) passive response under the ZD traces. The fitted values are: τ_*m*_ (ms) (using −30 pA TTX trace) = 32.8 (*Cell 1*), 29.1 (*Cell 2*), and 40.5 (*Cell 3*). τ_*m*_ (ms) (using −120 pA ZD trace) = 32.3 (*Cell 1*), 33.8 (*Cell 2*), and 39.3 (*Cell 3*). *Cell 1* in particular exhibited a very good match between the −30 pA TTX and −120 pA ZD traces. We also fitted the membrane time constant for the models, using a −120 pA current clamp step in the models without *I*_*h*_ included. Resulting *V*_*m*_ traces were fit in the same way as the experimental traces, except that the *V*_*m*_ data points were weighted by the relative time step of integration in the NEURON simulations such that data points in the *V*_*m*_ vector closely spaced in time would be weighed less. This ensured that the fit was not disproportionately weighed by the early, rapidly changing charging portion with many more data points. The fitted values are: τ_*m*_ (ms) (passive model) = 30.4 (*Cell 1*), 27.4 (*Cell 2*), and 36.4 (*Cell 3*).

### Mathematical Equations for the Current Due to H-Channels

The specification of the current for h-channels, *I*_*h*_, was taken from our previous work (Lawrence et al., 2006; Sekulić et al., 2014). However, the kinetics for activation and deactivation, the steady-state activation curves, and the conductance densities were defined on a per-cell basis in the present work. This required moving the relevant variables in the *I*_*h*_ MOD-file into the PARAMETER block to allow per-cell configuration in the NEURON code.

The conductance-based mathematical formulation used to represent current flow through h-channels is given by:

(1) $$\begin{array}{r} I_{h}=G_{h}\cdot r\left(V-E_{h}\right) \end{array}$$

(2) $$\begin{array}{r} \frac{dr}{dt}=\frac{r_{\infty }-r}{\tau_{h}} \end{array}$$

(3) $$\begin{array}{r} r_{\infty }=\frac{1}{1+\exp\left(\frac{V-V_{1/2}}{k}\right)} \end{array}$$

where *G*_*h*_ is the maximal channel conductance for the h-channels, *r* is the activation variable, *E*_*h*_ is the h-channel reversal potential, *r*_∞_ is the steady-state activation, *k* is the slope of activation and *V*_1/2_ is the potential of half-maximal activation of *I*_*h*_, τ_*h*_ is the time constant of activation, *V* is the membrane potential, and *t* is time. The voltage dependence of τ_*h*_ is given by a double exponential expression with parameters *t*_1_, *t*_2_, *t*_3_, *t*_4_, *t*_5_ as follows:

(4) $$\begin{array}{r} \tau_{h}\left(V\right)=\frac{1}{\exp\left(-t_{1}-t_{2}V\right)+\exp\left(-t_{3}+t_{4}V\right)}+t_{5} \end{array}$$

### Extraction of H-Channel Characteristics From Voltage and Current Clamp Traces

Given our experimental protocol (see Table 1), we were able to obtain h-channel current (*I*_*h*_) reversal potentials, activation kinetics, and steady-state activation for each of the three chosen cells.

#### Reversal Potential

To obtain the reversal potential for *I*_*h*_, we first removed the leak components and capacitive transients from the voltage clamp recordings in order to isolate the *I*_*h*_ components. This was done by taking the traces obtained by the reversal potential protocol (step #6 in Table 1) and subtracting from them the capacitive response generated by an equivalent magnitude voltage clamp deflection from the *I*_*h*_ activation protocol with ZD7288 application (step #8 in Table 1), resulting in *I*_*h*_ tail currents (see Supplementary Figure 2). The traces were then smoothed using the *rloess* smoothing function in MATLAB, which performs local linear regression over a window, using weighted linear least squares. The smoothing window was set to 25 ms so that only noise in the recordings was removed, and not time-dependent changes attributable to ion channel currents.

To create the current-voltage (I–V) plot, a fixed time point after the capacitive transient ended was determined by eye, which allowed us to obtain the time point of maximum deflection after the voltage clamp step (Magee, 1998; Molleman, 2003). We refer to this as the “fixed” time point for determining the I–V plot. The validity of this technique relies on the assumption that the maximal number of h-channels are still open by the time the capacitive transient is abolished, so that the resulting current does not depend on changes in the conductance, only on the driving force. The fact that *I*_*h*_ deactivates slowly means that this assumption is likely to be a safe one. However, to account for the possibility of early channel closure, a second method for extracting the current values and constructing an I–V plot was used. This consisted of fitting single exponential functions to the time course of the decay of current upon the step relaxation of the voltage clamp, which is used primarily to determine the voltage-dependent time constants of deactivation. The fitted exponential functions were then evaluated at the time of the relaxation of the voltage clamp step. In this way, we could deduce the amount of current that is masked by the capacitive transient by extrapolating the value from the exponential functions that were fitted on the non-capacitive portions of the current trace. That is, the functions were fitted to a window corresponding to the fixed point as the start time, and the end of the voltage clamp step as the end time. We refer to the current values and resulting I–V plot as the “extrapolated” method. We note that the smoothed traces were only used for the fixed method so that noisy fluctuations in the current traces did not unduly influence the resulting I–V plot; however, for the extrapolated method the exponential functions were fitted using the original, non-smoothed subtracted traces. The current traces with fitted exponentials are shown in Supplementary Figure 2, and I–V plots for both fixed and extrapolated methods are shown in **Figure 2B**. The resulting reversal potential (*E*_*h*_) values for each cell were determined by fitting a first-order polynomial to the linear portion of the I–V curve only. For *Cell 1* and *Cell 2*, the linear portion of the extrapolated I–V curve overlapped with the fixed I–V curve, and the resulting *E*_*h*_ values were similar between the two methods. For these cells, we therefore took *E*_*h*_ from the extrapolated I–V curves. For *Cell 3*, however, the capacitive transients disrupted the response and affected the fitting so that the extrapolated I–V values did not exhibit as strong of a linear relationship as the fixed I–V values. One possible explanation for the distorted (non-linear) measurements of I–V values with *Cell 3* is that the current traces for *I*_*h*_ deactivation, from which the reversal potential I–V plots were determined, did not match fully between the control case (with only TTX/4-AP/TEA blockers) and the later protocol with the *I*_*h*_ blocker ZD7288, due to the effects of noise. Thus, the subtraction of the two to remove the leak components introduced some distortion in the resulting current traces. As a result, although the resulting *E*_*h*_ values between extrapolated and fixed points were similar, we took *E*_*h*_ for *Cell 3* from the fixed I–V curve instead, to minimize possible error from using the line fitted with only 4 out of the 8 possible I–V datapoints (**Figure 2B**, *Cell 3*). The resulting *E*_*h*_ values for all cells are given in **Table 3**. These values are in general agreement with literature values of *I*_*h*_ reversal potentials in OLM cells (Maccaferri and McBain, 1996).

#### Voltage-Dependent Time Constant of Activation and Deactivation and Steady-State Activation

To obtain the time constants of activation/deactivation for *I*_*h*_ (τ_*h*_) we used the recordings where a voltage clamp protocol with an initial clamp at a holding potential was then stepped to various hyperpolarized potentials, measuring the resulting transmembrane current (#3 in Table 1). The identical protocol was then performed with the *I*_*h*_-specific blocker ZD7288 (#8 in Table 1). Using this data, we subtracted the ZD7288 traces from the control traces to isolate *I*_*h*_ for the activation time constants. Then, single exponential functions were fitted to the time-varying change in current upon each voltage step. *I*_*h*_ showed no voltage-dependent inactivation. To obtain the deactivation time constants, single exponential functions were fitted to the tail currents as obtained above (in the Reversal potential section). To construct the curve of voltage-dependent activation and deactivation kinetics, the time constants of activation were combined with the deactivation time constants obtained from the tail currents (see Supplementary Figure 2).

The time course of activation and deactivation was then described using a double exponential function of the form given by Equation (4) in section Methods, with parameters *t*_1_, *t*_2_, *t*_3_, *t*_4_, *t*_5_ to be fit. Fitting of the double exponential functions was done using a nonlinear least squares fit in MATLAB. However, due to the method of leak subtraction, some noisy differences in the capacitive transients between the two traces used in the subtraction may have overlapped with the initially observable *I*_*h*_ deactivation kinetics in the short time window upon current clamp release (see Supplementary Figure 2A). Accordingly, many of the deactivation time points were clear outliers and thus not reasonable measures of *I*_*h*_ deactivation, and these were not included in the fitting. All of the data points (including those not included in the fitting are shown in Supplementary Figure 3A). The resulting fitted values for the voltage-dependent time constant of activation and deactivation are given in Table 3 and plotted in **Figure 2C**. We note that the shape of the time constant of activation function is roughly similar across the three cells, with particular overlap between *Cell 1* and *Cell 2*. In all three cases, the slowest component of the time constant activation function is around 300 ms, whereas the fast component is less than 100 ms for all three cells.

#### Steady-State Activation

The steady-state activation curves, *r*_∞_, for the OLM cells were constructed by measuring the current amplitude in the ZD7288-subtracted traces at the end of each step of the voltage clamp protocol for *I*_*h*_ activation (see Supplementary Figure 2). The current at each voltage step was plotted and normalized to the greatest recorded current value which for h-channels is at the most hyperpolarized range. Then, a Boltzmann function for *r*_∞_ (Equation 3) with parameters *V*_1/2_ for the voltage at half-activation and slope factor *k* for the steepness of the sigmoidal curve, was fitted to each cell's voltage-dependent activation data. The fitted values are given in Table 3, and the resulting activation curves for the three cells are shown in **Figure 2D**.

#### Maximal Conductances

To determine the maximal conductance for *I*_*h*_, *G*_*h*_, we used the tail currents from the reversal potential step protocol as this corresponded to the point in time when *I*_*h*_ was fully activated (Magee, 1998; Dougherty et al., 2013). These currents were thus measured when all h-channels are opened, and thus describe the ratio of maximum current to voltage needed to obtain I-V plots for determining *G*_*h*_ (Molleman, 2003). The slope of the linear portion of the I–V plot for the tail currents, with the reversal potential as origin (denoting zero current flow), was used as the measure of *G*_*h*_. As described above, a line was fitted to the linear portion of the I–V plots for all three cells to determine the reversal potential. The slope of the line gives *G*_*h*_ and the resulting values for the three cells are given in Table 3. When scaled by the surface area, we obtain an *G*_*h*_ as a conductance density that is used in the model code.

### Full Spiking Multi-Compartment Model Optimizations

In creating full spiking models, we used the final passive model backbone with h-channels in the dendrites, and used the same complement of ion channel types that had been used in previous instantiations of the OLM cell model (Lawrence et al., 2006; Sekulić et al., 2014). The equations used are all given in the Appendix of Lawrence et al. (2006). They include transient sodium, fast and slow delayed rectifier potassium, A-type potassium, M-type, T- and L-type calcium, and calcium-dependent potassium channels. Their conductances in soma (*s*), axon (*a*), or dendrites (*d*) are represented respectively as *G*_*NaT*_, *G*_*Kdrf*_, *G*_*Kdrs*_, *G*_*KA*_, *G*_*M*_, *G*_*CaT*_, *G*_*CaL*_, *G*_*KCa*_ as given in Supplementary Table 2.

In our optimizations, we allowed *G*_*NaT*_, *G*_*Kdrf*_, *G*_*Kdrs*_ to vary independently in the soma, dendrites, and axon, and we also allowed the sodium channel to have some flexibility by allowing alterations in its voltage dependency, i.e., introducing a free parameter, *V*_*shift*_ that could change by ± 7 mV. Note that soma, dendrites, and axon each have an independent *V*_*shift*_ parameter, but the *V*_*shift*_ value remains the same across forward and backward rate activations and inactivations such that activation and inactivation curves shift by the same amount and the “activation/inactivation window” stays constant. Except for the inclusion of *V*_*shift*_, the activation and inactivation equations underlying the sodium current are the same as used previously (Lawrence et al., 2006), and as based on experimental data of Martina et al. (2000). For completeness, the equations for the sodium current, *I*_*NaT*_, are shown below:

(5) $$\begin{array}{r} I_{NaT}=G_{NaT}\cdot m^{3}h\left(V-E_{h}\right) \end{array}$$

(6) $$\begin{array}{r} \frac{dm}{dt}=\alpha_{m}\left(1-m\right)-\beta_{m}m \end{array}$$

(7) $$\begin{array}{r} \frac{dh}{dt}=\alpha_{h}\left(1-h\right)-\beta_{h}h \end{array}$$

where, for somatic compartments,

(8) $$\begin{array}{r} \alpha_{m}\left(V\right)=\frac{-0.1\left(V+38-V_{shift}\right)}{\exp\left(-\left(V+38-V_{shift}\right)/10\right)-1} \end{array}$$

(9) $$\begin{array}{r} \beta_{m}\left(V\right)=4\exp\left(-\left(V+63-V_{shift}\right)/18\right) \end{array}$$

(10) $$\begin{array}{r} \alpha_{h}\left(V\right)=0.07\exp\left(-\left(V+63-V_{shift}\right)/20\right) \end{array}$$

(11) $$\begin{array}{r} \beta_{h}\left(V\right)=\frac{1}{1+\exp\left(-\left(V+33-V_{shift}\right)/10\right)} \end{array}$$

and for dendritic and axonal compartments,

(12) $$\begin{array}{r} \alpha_{m}\left(V\right)=\frac{-0.1\left(V+45-V_{shift}\right)}{\exp\left(-\left(V+45-V_{shift}\right)/10\right)-1} \end{array}$$

(13) $$\begin{array}{r} \beta_{m}\left(V\right)=4\exp\left(-\left(V+70-V_{shift}\right)/18\right) \end{array}$$

(14) $$\begin{array}{r} \alpha_{h}\left(V\right)=0.07\exp\left(-\left(V+70-V_{shift}\right)/20\right) \end{array}$$

(15) $$\begin{array}{r} \beta_{h}\left(V\right)=\frac{1}{1+\exp\left(-\left(V+40-V_{shift}\right)/10\right)} \end{array}$$

#### Optimization Approach and Parameter Details

For the optimizations, we did the following:

1. Performed multi-objective optimizations using the BluePyOpt module in Python (Van Geit et al., 2016) and high performance computing resources via the Neuroscience Gateway (Sivagnanam et al., 2013) to find ion channel conductances in order to minimize the error across multiple features in the electrophysiology (see Supplementary Table 3).
2. Fine-tuned the parameter ranges and objectives to avoid areas of the parameter space that generate undesirable results and keep re-doing the optimizations using this approach until the top models consistently generate appropriate electrophysiologies. The parameter ranges used that produced the final models are shown in Supplementary Table 2.

During the optimizations, the fitness for each model is quantified as the sum of the number of standard deviations away from the experimental target efeature values and as such, is a unitless quantity. Standard deviation values (Supplementary Table 3) are chosen manually in order to weight each efeature (i.e., since we are fitting each model to a single voltage trace, there is no standard deviation that can be derived from the experimental data). Note that because the standard deviation values are chosen manually, choosing smaller standard deviation values to weight specific efeatures will increase the magnitude of the fitness values since this increases the number of standard deviations away from the target value. As such, since we used small standard deviation values, this caused the fitness values of the model to artifactually be quite large. The top five optimized models for *Cell 1* and *Cell 2* are presented in **Figure 6A** and Supplementary Figure 4. Their fitness values are: (*Cell 1:* 411.53, 425.38, 430.68, 430.93, 438.65; *Cell 2:* 660.96, 665.49, 669.06, 678.58, 684.60).

In order of model rankings (i.e., [1*st*,.,5*th*]), the values below are the *V*_*shift*_ parameters (in mV) for the top five full spiking models (see **Figure 5C** to see the resulting voltage-dependencies).

*Cell 1*:

*V*_*shift,s*_ = [−4.83, −6.55, −6.70, −6.46, −6.68],

*V*_*shift,d*_ = [4.85, 6.37, 3.71, 2.86, 4.17],

*V*_*shift,a*_ = [2.49, 2.82, 2.84, 5.70, 2.82].

*Cell 2*:

*V*_*shift,s*_ = [−4.36, −4.69, −4.88, −4.36, −4.36],

*V*_*shift,d*_ = [−1.26, −1.12, −0.54, −1.46, −1.41],

*V*_*shift,a*_ = [6.57, 6.57, 6.30, 6.57, 6.57].

After performing several optimizations and adjusting the parameters to improve the optimization outputs, we used the following optimization parameters for both models: Number of Offspring = 100, Number of Generations = 200, Mutation Rate = 0.15, Crossover Rate = 0.85, Eta (i.e., learning rate) = 0.5, Optimizer = “IBEA,” Random Seed = 61 (*Cell 1*) and 9 (*Cell 2*).

All of the objective features that were used in the optimization are listed in Supplementary Table 3, and the parameter ranges are given in Supplementary Table 2. Features 1–10 were used for the +30, +60, and +90 pA current injection protocols. Features 11–12 were only used for the +60 and +90 pA current injection protocols, since the +30 pA current injection did not always generate a sufficient number of spikes for those features to be calculated. Since we were fitting the models to single current injection traces, standard deviation values were chosen manually for each objective feature, in order to weight each objective feature by hand. Since standard deviation is used in computing the fitness for each model (i.e., fitness is quantified as the sum of the number of standard deviations away from the experimental target efeature values), manipulating these values offered a way to weight particular target measurements. More specifically, we initially chose standard deviations that were 1–2 order of magnitudes smaller than the largest significant digit for each measurement. For example, *AP*_*duration*_*half*_*width* in the somatic area of a neuron is usually a small value between 0.5 and 2 ms, and we used a standard deviation of 0.01 ms for this efeature. If the optimization ended up under-performing on any specific efeature measurements, we would sometimes attempt to improve it by using smaller standard deviation values for those measurements. Though this had some mild effects on improving the optimizations, constraining the free parameter ranges ended up showing much better improvements in the optimization results. We also added a heavy penalization on models that generated spikes during the baseline periods. Finally, in order to make BluePyOpt compatible with the OLM cell model compartmentalization, we adjusted BluePyOpt's method for compartmentalization such that it uses the *d*_λ_ rule (Hines and Carnevale, 2001).

To check if axonal properties were appropriate for what is known experimentally (Martina et al., 2000), we performed simulations with our final optimized spiking models of *Cell 1* and *Cell 2* to investigate morphological sites of action potential (AP) initiation. Specifically, Martina et al. (2000) previously showed that depending on whether a short high-intensity current or a long low-intensity current was injected into the soma, an AP would occur initially in the soma or axon-*bearing* dendrite, respectively. For both models of *Cell 1* and *Cell 2*, short high-intensity current evoked action potential initiation in the soma, but long low-intensity current evoked action potential initiation in axon-*lacking* dendrites. This suggests that specialized distributions of spike-initiating channels are missing in the axon of the model and are necessary for correctly setting the action potential initiation site. Given that OLM cell axonal channel properties are unknown, we did not venture further into specializing axonal properties in our models.

## Results

### YFP-Positive Stratum Oriens Interneurons From SOM-Cre/Rosa26YFP Mice Contain a Population of OLM Cells

**Figure 1:**
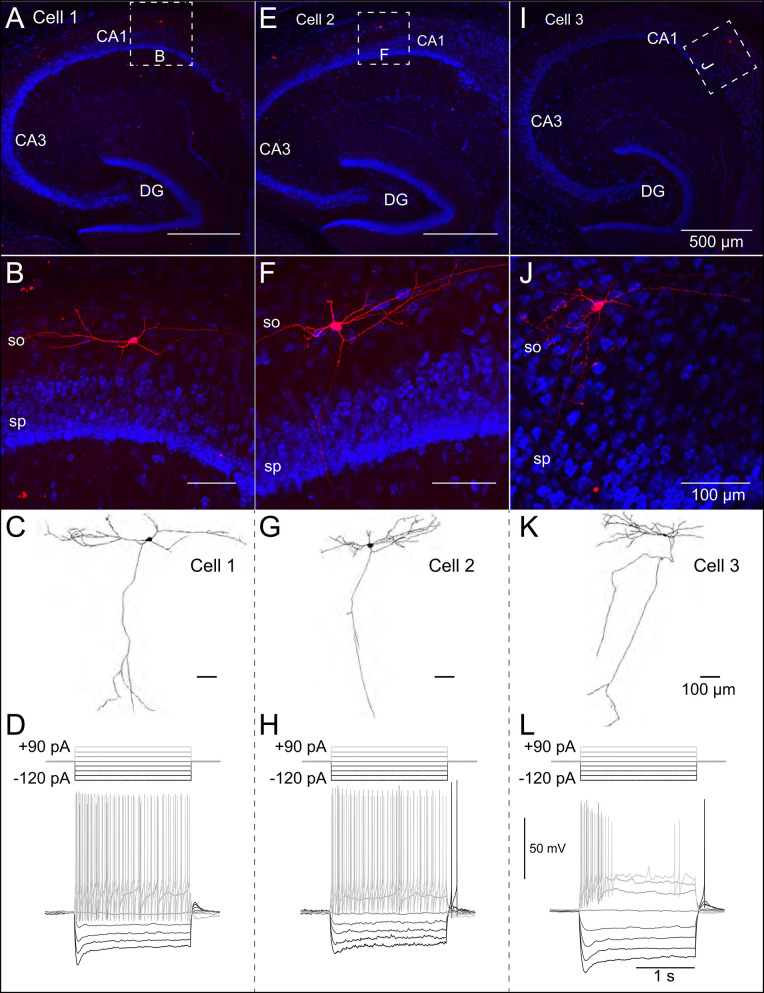
Morphological and electrophysiological properties of OLM interneurons. Anatomical and electrophysiological properties of three OLM cells: **(A–D)** (*Cell 1*), **(E–H)** (*Cell 2*), and **(I–L)** (*Cell 3*) are shown. **(A)** Low magnification confocal image of the hippocampus showing *Cell 1* localized within CA1. Dashed square in **(A)** indicate boundaries of higher resolution images in **(B)**. **(B)** Expanded view of *Cell 1* localized to the stratum oriens layer of CA1. **(C)** Reconstructed morphology of *Cell 1*, **(D)** Voltage responses to a family of 2 s-long current hyperpolarizing (black; −90, −60, −30 pA) and depolarizing (light gray; +90, +60, +30 pA) current steps from −73 mV for *Cell 1*. Synaptic blockers were present (see section Methods). Hyperpolarization-induced sag is evident upon introduction of the −90 pA current step. *Cell 2* and *Cell 3* are shown in **(E–H,I–L)**, respectively.

Patch-clamp recordings from 45 SOM-YFP neurons in the stratum oriens of SOM-Cre/Rosa26YFP mice were obtained using a detailed protocol as given in Table 1. Of these recordings, 11 of them met criteria for stability (see section Methods) and were morphologically confirmed as OLM interneurons, having horizontal cell body and dendrites confined to the oriens layer, and perpendicularly projecting axons which ramify in the lacunosum/moleculare layer. An analysis of these 11 cells is given in Table 2 in which the experimental data analysis was performed as in Yi et al. (2014). From these 11 OLM cells, three (*Cell 1, Cell 2, Cell 3*) were chosen for subsequent detailed analyses and computational model creation. Their specific properties are given in Table 2. Morphological and electrophysiological characteristics for these three OLM cells are shown in Figures 1A–L.

**Table 2:** Passive and active properties of OLM cells from SOM-Cre/Rosa26YFP mice.

**Property**	**SOM-YFP (*n* = 11)**	**Cell 1**	**Cell 2**	**Cell 3**
*R*_*in*_ (MΩ)	314.3 ± 33.8	360.1	259.0	490.2
*C*_*m*_ (pF)	107.1 ± 13.0	62.8	123.7	79.6
Sag ratio (SS/peak)	0.89 ± 0.03	0.70	0.84	0.74
τ_*m*_ (ms)	31.1 ± 2.5	22.6	32.0	39.0
*I*_*hold*_ (pA)	3.4 ± 4.8	2.7	−2.8	2.5
First AP half-width (μs)	595.1 ± 26.4	617.2	551.2	691.6
First AP height (mV)	66.0 ± 2.7	69.3	61.4	67.7
Adaptation coefficient	0.5 ± 0.1	0.41	0.61	0.49
Frequency at 90 pA (Hz)	15.5 ± 2.1	20	19.5	8
Frequency at 60 pA (Hz)	8.3 ± 1.5	12.5	11.5	3
Frequency at 30 pA (Hz)	2.1 ± 0.8	4	1	1

*Values are presented as means ± SEM. R_in_, input resistance; τ_m_, membrane time constant; AP, action potential; C_m_, membrane capacitance; SS, steady state; I_hold_, the current injected to hold the recorded cell at −60 mV (not junction potential corrected). Passive properties were extracted at a current injection of −60 pA. Active properties were extracted at a current injection of 90 pA, unless otherwise specified. APs were detected at a derivative threshold of 20 mV/ms. The adaptation coefficient was calculated by dividing the first inter-spike interval by the last inter-spike interval of the AP train during current injection of 90 pA*.

SOM-YFP stratum oriens interneurons mice had slow membrane time constants and relatively high input resistances, in accordance with our previous study (Yi et al., 2014). Action potential half-widths were larger and membrane time constants were slower than previously reported for YFP neurons from PV-CRE/Rosa26YFP mice, consistent with the exclusion of PV-positive basket and bistratified cells from this population. Moreover, this population had considerable hyperpolarization-induced sag, which, when combined with their higher input resistance, is considered a hallmark feature of OLM cells (Maccaferri and Lacaille, 2003).

### Multi-Compartment OLM Cell Models Capture Corresponding Passive Responses

We used the NEURON simulation environment (Hines and Carnevale, 2001) to develop our multi-compartment models. Figures 1A–J shows representative confocal images of the three cells, with the reconstructed cell morphologies shown in Figures 1C–K, paired with electrophysiological OLM cell profiles featuring hyperpolarization-induced sag. Details of the model reconstructions are given in section Methods.

**Figure 2:**
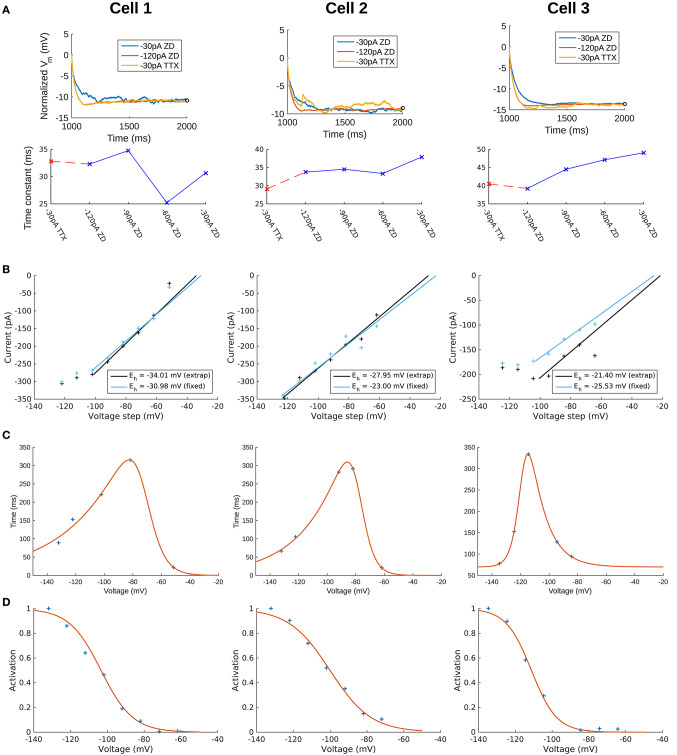
Extracted passive and *I*_*h*_ properties of OLM cells from experiment. **(A)** (Top) Membrane potential (*V*_*m*_) normalized at steady-state, showing noisier responses of −30 pA ZD trace compared to the −120 pA ZD trace. (Bottom) Fitted membrane time constants (τ_*m*_) for all current clamp steps with ZD7288 application, as well as the −30 pA TTX trace. “ZD traces” are those in which synaptic and voltage-gated currents are blocked including h-currents with ZD7288 application, and “TTX traces” are the same except that h-currents are not blocked (protocols #7 and #4, respectively in Table 1 of section Methods). **(B)** *I*_*h*_ reversal potential as determined from current-voltage obtained from tail currents. See section Methods for difference between “extrap” and “fixed” values in plots and further details. **(C)** Time constants (τ_*h*_) or kinetics of activation and deactivation as determined from voltage clamp experiments. **(D)** Steady-state activation curves (*r*_∞_) as determined from voltage clamp experiments. See section Methods for details.

To capture the passive response of the three cells we used the 2 s-long −120 pA current clamp traces in which all synaptic and voltage-gated channels were blocked. This choice was made because we found that the −30 pA traces were noisier in general (see Figure 2A, top panels), and the −120 pA traces best captured the passive response of the cells. This can be seen from a comparison of the membrane time constants (τ_*m*_) for different current clamp steps (see Figure 2A, bottom panels) and consideration of the protocol ordering of the recording session as in Table 1. Time constants were obtained by quantitatively fitting single exponential equations to the *V*_*m*_ responses from the time point of the onset of the hyperpolarization current clamp step (1,000 ms) to the point at which the steady-state of the *V*_*m*_ response was approximately achieved (Figure 2A, top panels). We refer to “TTX” and “ZD” traces as those with blockers given respectively by steps #4 and #7 in Table 1. For all cells, τ_*m*_ for the −30 pA TTX trace was most closely matched by the −120 pA ZD trace (dashed red line), with the subsequent ZD traces (−90, −60, and −30 pA) exhibiting an increased, and hence slower, membrane response (Figure 2A, bottom panels). The fact that the −120 pA ZD trace exhibited a similar response as a current injection of one quarter magnitude and without h-channels being blocked (i.e., −30 pA TTX) indicated that in both cases, the response of the membrane was mostly passive. Thus, given the lower signal-to-noise of the −30 pA ZD traces, we considered that the passive properties obtained using the −120 pA ZD traces would be better representations of the electrotonic features of the experimental cells. Further details are provided in section Methods.

To confirm that the −120 pA ZD traces led to better fits of the cells' passive properties, we compared the fits obtained using −120 and −30 pA ZD traces. The resulting fitted passive parameters of axial resistivity (*R*_*a*_), specific capacitance (*C*_*m*_), leak conductance (*G*_*pas*_), and leak reversal potential (*E*_*pas*_) are displayed in Supplementary Table 1. For each cell, the cumulative root-mean-square error (RMSE) across all traces used for each fit was lower when the −120 pA ZD trace was used for fitting the passive properties (Supplementary Table 1, left column for each cell). These parameters with the respective cell morphologies form the “backbone” of the OLM cell models, and there was a favorable comparison of input resistances and time constants between model and experiment (see section Methods for details). We noted that the *C*_*m*_'s obtained from our model fits were lower than the ≈ 0.9–1μF/cm^2^ that have been previously reported as a “standard” value in mammalian neurons (Gentet et al., 2000). To consider potential errors in dendritic estimations due to tissue shrinkage, swelling and other limitations, we did a simple dendritic scaling check as described in Appendix 1 (Supplementary Material), that provided support for these low capacitance values.

### OLM Cell Multi-Compartment Models With Constrained Passive Properties and Added H-Channel Models Do Not Match Experimental Recordings

We use a standard conductance-based formalism to represent the ionic current due to h-channels (*I*_*h*_), and we obtain *I*_*h*_ parameter values as fits to the experimental data for each of the three cells (see section Methods). The parameters extracted from each of the OLM cells in this work included the *I*_*h*_ reversal potential (*E*_*h*_), the time constants of activation and deactivation of *I*_*h*_ (τ_*h*_), the steady-state activation curve of *I*_*h*_ (*r*_∞_), and the *I*_*h*_ maximum conductance density (*G*_*h*_). They are plotted in Figures 2B–D and parameter values are shown in Table 3. These h-channel models are added to our OLM cell multi-compartment models' “backbone” of morphological reconstructions and passive property fits.

**Table 3:** Computed *I*_*h*_ parameter values obtained from fits to experimental data with computed conductance densities for somatic or somatodendritic distributions.

**Parameter**	**Cell 1**	**Cell 2**	**Cell 3**
*E*_*h*_ (mV)	−34.0	−27.9	−25.2
*V*_1/2_ (mV)	−103.4	−100.1	−111.3
*k* (slope factor)	8.63	11.16	6.88
*t*_1_ (ms)	8.03	8.98	35.09
*t*_2_ (ms)	0.025	0.035	0.24
*t*_3_ (ms)	−4.40	−8.49	−4.28
*t*_4_ (ms)	0.15	0.19	0.088
*t*_5_ (ms)	7.32 × 10^−6^	3.57 × 10^−7^	69.72
Total *G*_*h*_ (nS)	4.17	3.64	2.20
*G*_*h*_ (pS/μm^2^), *H*_*dist*_ = 0	0.546	0.279	0.380
*G*_*h*_ (pS/μm^2^), *H*_*dist*_ = 1	0.142	0.104	0.120

**Figure 3:**
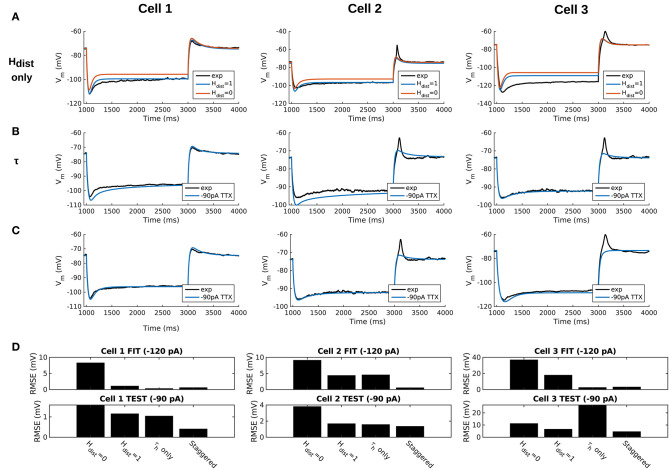
Staggered re-fitting of *I*_*h*_ and passive property parameters successfully captures experimental cell responses to current clamp steps. **(A)** Boundary conditions of *H*_*dist*_ parameter [0, 1] showing inappropriate fits when putting all experimentally derived parameters together with fitted passive “backbone.” **(B)** Re-fitting only τ_*h*_ does not provide good fits (*H*_*dist*_ = 1). **(C)** Staggered re-fitting of parameters results in good fits. Shown in **(B,C)** are −90 pA TTX traces which are “test” traces not used for fitting (−120 pA TTX trace was used for fitting, not shown). **(D)** Root-mean-square error (RMSE) of model responses to experimental traces in the case of traces used for fitting (top) and those for validation (bottom).

We demonstrated in our previous work that OLM cell models exhibited a tradeoff between total membrane *G*_*h*_ and the dendritic distribution of h-channels so that if the total *G*_*h*_ was conserved, the resulting model output would be appropriate (Sekulić et al., 2015). Now, for the first time, we have a measure of total *G*_*h*_. Thus, a key prediction for the resulting multi-compartment models is that the total *G*_*h*_ will constrain the distribution of h-channels to allow the models to appropriately capture the OLM cell electrophysiological characteristics. To consider this, we added an additional parameter to our models termed *H*_*dist*_, which is defined as the centripetal extent for which h-channels are inserted in the dendrites. It is defined by a real-valued number in the range of [0, 1] and represents the fraction of maximum dendritic path length from the soma on a per-cell basis. Compartments with a path length from the soma that was smaller than any given *H*_*dist*_ value were included when subsequently inserting h-channels, whereas those compartments whose distance from soma exceeded *H*_*dist*_ were excluded. The boundary condition of *H*_*dist*_ = 0 is defined as the case where h-channels are only present in somatic compartments and not present in the dendrites. A non-zero value for *H*_*dist*_ meant that the amount of dendrite specified by *H*_*dist*_ itself had h-channels in addition to the somatic compartments. *H*_*dist*_ = 1 refers to full somatodendritic presence of h-channels, i.e., uniform distribution in the dendrites and soma. The per-cell *I*_*h*_ parameters were inserted into each of the three models and two cases of *H*_*dist*_ were initially considered to test the boundary cases: either no dendritic h-channels (*H*_*dist*_ = 0) or full, uniform distribution of h-channels in the dendrites (*H*_*dist*_ = 1). The resulting h-channel conductance density was calculated by dividing total *G*_*h*_ by the resulting surface area of only somatic or somatodendritic compartments. These values and the other h-channel parameters are given in Table 3. The model outputs were fitted to experimental traces similarly as in the case with passive properties and ZD traces (see section Methods). The *V*_*m*_ output of each of the three models being developed here, with boundary conditions of *H*_*dist*_ = 0 or 1 for the case of −120 pA current clamp injection, compared to the experimental TTX trace for each cell, is shown in Figure 3A. It is clear that these models do not fully match the experimental traces. Although we did explore *H*_*dist*_ values that were between 0 and 1 (not shown), it is clear that given the fits shown in Figure 3A, it is unlikely that changing *H*_*dist*_ to a value between 0 and 1 would improve the fits to the experimental data.

### A Staggered Re-fitting Procedure Yields Consistent and Generalized Model Fits for OLM Cells With Dendritic H-Channels

Given the sub-optimal match of our models with the experimental data, even with model parameters determined from experiment on a per-cell basis, we considered the possibility that one or more of the parameters were mismatched between the experimental cells and the parameter values derived from the recordings. We considered re-fitting the various parameters in the model to ensure that *I*_*h*_ and passive parameters resulted in correct output for each cell. However, due to the sheer number of parameters present in the model, care needed to be taken in how the parameters were adjusted as there are many interdependencies between the fitted parameters. For instance, when *I*_*h*_ is present, the trajectory of the *V*_*m*_ response upon a step of hyperpolarizing current in a cell depends not just on *C*_*m*_ and *R*_*a*_, but also on the time constants of activation and deactivation of h-channels (τ_*h*_) and, to a degree, the h-channel steady-state activation curve (*r*_∞_). Therefore, if there is error in the model *V*_*m*_ response compared to the experimental trace in this portion of the trace (Figure 3A), the mismatch between model and experiment may have been either due to the passive parameters, or due to τ_*h*_ or *G*_*h*_, which gated by the activation, determines the amount of *I*_*h*_. The problem, then, is how to attribute errors in any particular portion of a *V*_*m*_ trace to any given parameter in the model.

We noted that the initial mismatch in the case of *H*_*dist*_=1 and for *Cell 1* and *Cell 2* seem primarily to be located in the initial hyperpolarizing phase and the sag portion (see Figure 3A). Because the τ_*h*_ functions were constructed using a limited set of data, it was reasonable to suppose that a large source of mismatch in this portion of *V*_*m*_ could be due to errors in the τ_*h*_ function itself. We thus re-fitted the parameters for τ_*h*_, namely *t*_1_, *t*_2_, *t*_3_, *t*_4_, *t*_5_ for all three cells, against each respective −120 pA TTX trace and then compared the models' responses to the other current clamp steps to see how much of the error could be accounted for by re-fitting τ_*h*_ alone Figure 3B shows how the −120 pA τ_*h*_ re-fit parameters compares to the −90 pA TTX trace for each cell. This re-fitting of τ_*h*_ alone could not address the mismatch in *V*_*m*_ between model and experiment although it may have played some role, as evidenced by improving the match in *V*_*m*_ in some cases. Thus other parameter re-fitting needed to be considered.

We adopted the following approach and rationale. Since the passive properties were not as tightly constrained as the *I*_*h*_ properties, and could account for some of the mismatch in both the transient and steady state portions of the traces, we re-fitted them first. That is, *R*_*a*_, *C*_*m*_, *G*_*pas*_, *E*_*pas*_. Turning to the *I*_*h*_ properties, we first re-fitted the total *G*_*h*_, which determines the per-compartment conductance density, as well as the steady-state activation curve *r*_∞_ since it determines the voltage dependency of how many channels are open. We could not fit *G*_*h*_ and *r*_∞_ in the reverse order because any error in *G*_*h*_—that is, how *I*_*h*_ scales with voltage when all channels are opened—could be accounted for by refitting *r*_∞_ by “flattening” it, thus lowering the total number of channels that are open at any given voltage. This would not be physiologically correct since the model would then imply that *I*_*h*_ is never fully activated, i.e., *r*_∞_ does not reach unity. Thus, by re-fitting *G*_*h*_ first, followed by *r*_∞_, we increased the likelihood that *r*_∞_ did not diverge too much from the experimental data points obtained from the protocol for *I*_*h*_ activation. Finally, we re-fitted τ_*h*_. If the passive properties and steady-state *I*_*h*_ due to *G*_*h*_ and *r*_∞_ accounted for much of the mismatch in *V*_*m*_, then the last step of re-fitting τ_*h*_ should allow for any mismatch due to τ_*h*_ to be corrected for.

Using this approach, which we termed a “staggered” re-fitting, we show the model outputs in Figure 3C where only the −120 pA TTX traces were used for fitting the parameters, with the −90 pA TTX traces provided test data to validate the fits. The results with this approach were clearly more successful than the previous approaches, as shown by the errors in Figure 3D. By fitting the parameters in such a way that the ones most likely to be responsible for errors in particular portions of the mismatched model *V*_*m*_ traces were fitted first, the resulting fits were now generalizable relative to the case of either re-fitting the passive properties alone, or re-fitting τ_*h*_ alone, since the staggered re-fitted values were able to match all of the other current clamp traces that were not used for fitting. Figures detailing this as well as considerations of overfitting, especially the pitfall of simultaneously adjusting all model parameters, are provided in Appendix 2 (Supplementary Material).

**Figure 4:**
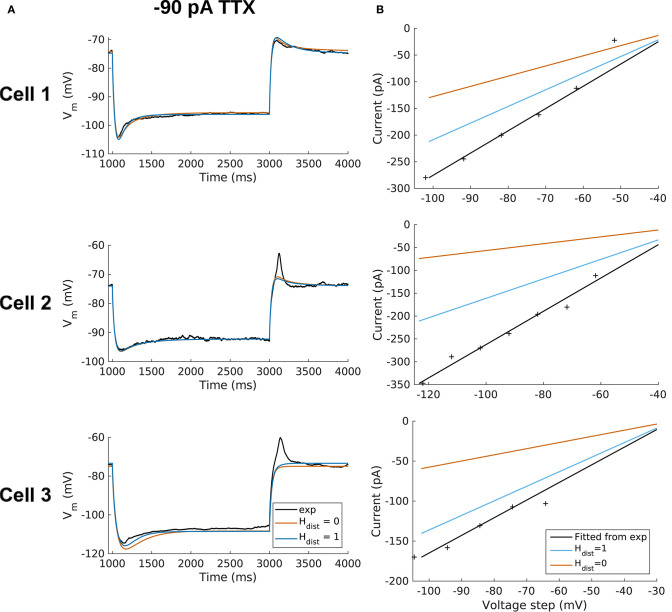
OLM cell models with somatodendritic h-channels, but not somatic only, appropriately capture experimental data. **(A)** Using a staggered re-fitting, both *H*_*dist*_ = 0 or 1 are good fits to the experimental data. Note that the −90 pA TTX traces are “test” traces and were not used for fitting (−120 pA TTX used for fitting), *H*_*dist*_ = 0 or 1. **(B)** *H*_*dist*_ = 1 is clearly more appropriate than *H*_*dist*_ = 0 relative to the experimental data as shown in plotting I–V curves.

All the models in the staggered re-fit were done with *H*_*dist*_ = 1, because that value was the one that provided the closest fit to the experimental traces (Figure 3A) when only passive properties were fit to the *V*_*m*_ traces and *I*_*h*_ parameter values were obtained from the voltage clamp protocols. These staggered re-fitted values are shown in Table 4, *H*_*dist*_ = 1 column. Model parameters before staggered re-fitting can be found in Supplementary Table 1 (passive properties) and Table 4 (*I*_*h*_ properties). We examined whether using *H*_*dist*_ = 0 and applying our staggered re-fitting approach could also produce good, generalizable fits to the experimental data. The models with *H*_*dist*_ = 0 fitted the experimental *V*_*m*_ traces well in all four current clamp steps as it did for *H*_*dist*_ = 1, and we show the comparison to the −90 pA TTX trace for *H*_*dist*_ values of both 0 and 1 in Figure 4A, noting that the −120 pA TTX trace was used for the fitting. The staggered re-fitted values for *H*_*dist*_ = 0 are also shown in Table 4. From a comparison across Table 4 of parameter values for *H*_*dist*_ = 0, 1 and original *I*_*h*_ parameter values fit to the experimental data (see Table 3, it is clear that the re-fitted parameter values using *H*_*dist*_=0 are inappropriate, specifically due to total *G*_*h*_ differences (experimental total *G*_*h*_ values from Table 3 shown in bold in Table 4 for convenience). For the case of *H*_*dist*_ = 1, *G*_*h*_ values are reasonably close to what was measured directly from the I–V plot of the reversal potential experimental protocol, unlike *H*_*dist*_ = 0, which exhibited *G*_*h*_ values that were much less than half of the experimentally-derived values. Given that this parameter was taken from the slope of the I–V plot, the values from *H*_*dist*_ = 0, if correct, would imply that the recorded current values were double the “true” values in the cell. This is graphically depicted in Figure 4B. We deemed this unlikely, and concluded that the divergence in the re-fitted *G*_*h*_ with *H*_*dist*_ = 1 compared to the experimental case indicated a much more reasonable error. Model *I*_*h*_ time constants of activation (τ_*h*_) and steady-state activation curves (*r*_∞_) for *H*_*dist*_ = 0,1 are shown for comparison along with the experimental fits in Supplementary Figure 3, lending further support for *H*_*dist*_ = 0 being unlikely.

**Table 4:** Final fitted model parameters using either *H*_*dist*_ = 0 or *H*_*dist*_ = 1.

**Parameter**	**Cell 1**		**Cell 2**		**Cell 3**	
	***H*_*dist*_ = 0**	***H*_*dist*_ = 1**	***H*_*dist*_ = 0**	***H*_*dist*_ = 1**	***H*_*dist*_ = 0**	***H*_*dist*_ = 1**
*R*_*a*_ (Ωcm)	34.4	125.2	285.4	348.1	211.8	317.9
*C*_*m*_ (μF/cm^2^)	0.20	0.27	0.37	0.38	0.52	0.58
*G*_*pas*_ (S/cm^2^)	7.36 × 10^−6^	7.58 × 10^−6^	1.16 × 10^−5^	1.19 × 10^−5^	8.26 × 10^−6^	8.68 × 10^−6^
*E*_*pas*_ (mV)	−64.0	−64.6	−61.5	−61.8	−75.7	−76.1
*E*_*h*_ (mV)	−34.0	−34.0	−27.9	−27.9	−25.2	−25.2
*V*_1/2_ (mV)	−103.1	−103.7	−100.6	−99.6	−108.4	−113.8
*k* (mV)	9.99	9.99	9.93	9.99	9.39	9.99
*t*_1_ (ms)	12.30	8.56	12.73	11.28	36.77	41.84
*t*_2_ (ms)	0.063	0.029	0.071	0.056	0.25	0.29
*t*_3_ (ms)	−22.87	−6.91	−20.38	−19.28	−3.22	−4.03
*t*_4_ (ms)	0.39	0.18	0.36	0.34	0.084	0.089
*t*_5_ (ms)	0.026	4.35 × 10^−5^	3.54 × 10^−6^	0.006	5.69	4.29
Total *G*_*h*_ (nS)	1.91	3.12	0.75	2.14	0.77	1.82
(Expt) Total *G*_*h*_ Cell 1 = 4.17 nS						
(Expt) Total *G*_*h*_ Cell 2 = 3.64 nS						
(Expt) Total *G*_*h*_ Cell 3 = 2.20 nS						

Hence, the fact that it was possible to match the experimental *V*_*m*_ traces using both *H*_*dist*_ = 0 (Figure 4A) and *H*_*dist*_ = 1 (Figure 3C) did not mean that they were equally valid. The benefit of having directly measured experimental values representing *G*_*h*_, τ_*h*_, *r*_∞_ from the *same* cell meant that we could confidently state that models with *H*_*dist*_ = 0, though they fitted the *V*_*m*_ traces, were not appropriate models because they did not match the experimentally-derived values. Thus, only when h-channels were spread into the dendrites did we find models whose *V*_*m*_ responses matched the experimental traces and whose total *G*_*h*_ and other parameter values were in reasonable agreement with the experimentally measured values. We thus predict that the experimental cells in the dataset used here have h-channels expressed in their dendrites, with biophysical characteristics as given in Table 4, *H*_*dist*_ = 1.

### Optimized Full Spiking Models of OLM Cells Capture Responses to Current Step Stimuli

We have so far developed three multi-compartment models of OLM cells with fitted passive and *I*_*h*_ parameter values. The presence of h-channels in the dendrites of these models was found to be the most appropriate distribution given the experimental data. We now focus on two of the OLM cell models—*Cell 1* and *Cell 2*—and move forward to include a full repertoire of ion channel types as used in previous OLM cell models (Lawrence et al., 2006), thus creating full spiking models available for use in further studies.

To do this, we optimized the parameter values (see Supplementary Table 2 for parameters and ranges) to depolarizing steps of the particular cell, where most voltage-gated ion channels were expected to be activated. We used BluePyOpt (Van Geit et al., 2016) to perform multi-objective optimizations that provided sets of parameter values which generated appropriate OLM cell voltage output at +30, +60, and +90 pA depolarizing steps (#2 in Table 1), given specified features (see Supplementary Table 3). We note that our fits were done using the specific experimental data sets and not to a set of experimental data with variances associated with electrophysiological features. We further note that we did our fitting using holding currents in line with the experimental data (4 pA for *Cell 1* and −5 pA for *Cell 2*). Further details are provided in section Methods.

**Figure 5:**
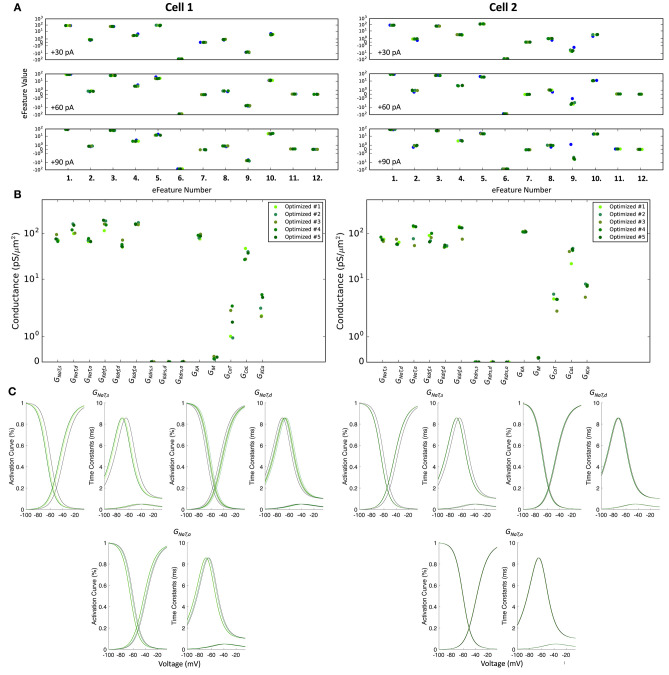
Optimized spiking model features and parameters. **(A)** Measurements of objective e-features for each of the top five optimized models (shades of green) during the +30, +60, +90 pA current injection steps. Each number on the x-axis corresponds to an e-feature. For corresponding e-feature names and descriptions, see Supplementary Table 3. The corresponding target values obtained from the experimental data are shown as blue dots. **(B)** Optimized conductance values in the top five spiking models. **(C)** Voltage-dependency of somatic, dendritic, and axonal sodium channels were allowed to shift during the optimizations. Here we show the resulting voltage-dependent activation curves and time constants in the top five spiking models (shades of green) as compared to the activation curve used in previous instantiations of the OLM cell model (black curves). See section Methods for specific numbers.

**Figure 6:**
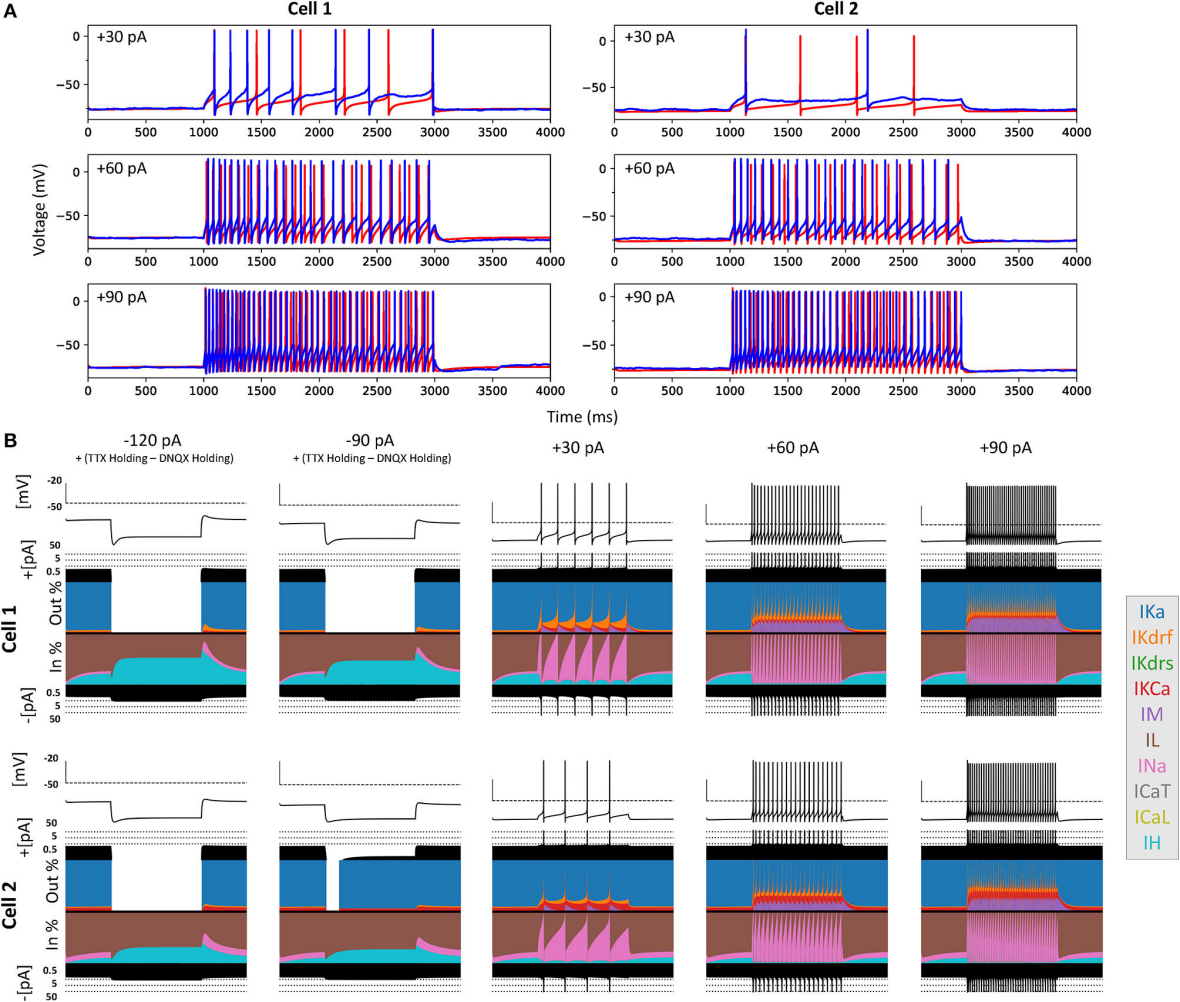
Full spiking optimized OLM cell models and underlying currents. **(A)** The most highly ranked optimized models for *Cell 1* and *Cell 2* are plotted in red, and the experimental data is plotted in blue. Model parameters were optimized using depolarizing +30, +60, and +90 pA current step recordings from protocol #2 given in Table 1. **(B)** Currentscapes for top spiking models. Using −120, −90, +30, +60, and +90 pA current injection steps, the currentscape plots (Alonso and Marder, 2019) indicate the relative current contributions (i.e., the color areas in the plots) of the total inward or outward channel currents (i.e., black areas at the top and bottom of each currentscape plot). Note that the recordings shown here are from the first dendritic compartment adjacent to the soma since calcium channels are not present in the somatic compartment. During hyperpolarizing steps, it is evident from these plots that *I*_*h*_ (IH) and the leak current (IL) are the primary contributors to the electrophysiological output. For depolarizing steps, we see the largest contributions are from A-type potassium current (IKa), fast delayed-rectifier current (IKdrf), sodium current (INa), and calcium-dependent potassium current (IKCa), with increasing contributions from M-type current (IM) as the current step magnitude gets larger. Slow delayed-rectifier current (IKdrs) contributes minimally.

The optimized spiking model features for the top five models relative to the experimental data are shown in Figure 5A, and the optimized parameter values are given in Figures 5B,C. Further details of the objective features and fitness values are provided in the Methods. The models with the resulting best fits are shown in Figure 6A, and the next four top fits are given in Supplementary Figure 4. Similar outputs were obtained in the top five ranked optimized models and all performed well in terms of capturing electrophysiological feature measurements (Figure 5A). *Cell 2* in particular had more difficulty with the “AHP_depth” electrophysiological feature, which is likely because the model failed to attain a high enough spike threshold, and thus the resulting “AHP_depths” were too low. While we tried to encourage the models to reach higher spike thresholds by allowing the sodium voltage dependencies to vary as free parameters in the optimizations (Figure 5C), in the end, the models could not fully capture the adaptation in spike threshold that was seen experimentally (i.e., the spike threshold appeared to increase during spiking at higher frequencies). These top models also had similar optimized parameter values (Figures 5B,C), though this may be a result of over-constraining the optimizations (see approach and parameter ranges in section Methods).

To ensure that the full spiking models did not affect the *I*_*h*_ fits, we applied hyperpolarizing steps to the full spiking models as done experimentally, and found that they were in full agreement with the experimental data, as shown in Supplementary Figure 5. It was expected that adding the full set of ion channel mechanisms would not affect the model's ability to match the hyperpolarizing steps since the additional currents are not active at these hyperpolarized values. This can be appreciated by looking at the contributions from the different currents at the different current steps using “currentscapes,” a novel visualization technique (Alonso and Marder, 2019). As shown in Figure 6B it is clear that only *I*_*h*_ and the leak current are active during the hyperpolarization steps, and not other ionic currents. In fact, contributions from all other currents during these hyperpolarization steps were minimized beyond being able to see them on the plots and outward current can become non-existent since the reversal potential for potassium is passed.

Taking advantage of our generated currentscapes (Figure 6B), we were able to easily observe several features in our optimized models. A prominent feature was the large contributions from A-type potassium currents during both the baseline periods as well as during spiking regime activities. Since we minimized slow delayed rectifier potassium conductances on purpose in order to achieve better fits (see section Methods and Figure 5), it was not surprising that the major contribution of outward currents during spikes was from fast delayed rectifier potassium ones. However, it was perhaps surprising that M-type and calcium-activated potassium currents provided such large contributions to outward currents, despite having considerably smaller conductances relative to the other outward ion channel types (Figure 5). Particularly, M-type exhibited larger current contributions during the after-hyperpolarization periods (AHP) at higher spike rates. In terms of inward current contributions, we did not see any observable contributions from the L-type and T-type calcium channel types. Mostly, inward current contributions in the spiking regimes were from sodium channels. However, *I*_*h*_ provided some observable contributions during the spike recovery periods, and also provided a larger contribution leading up to the first spike.

We note that our goal was to obtain spiking models that could adequately capture the data for the particular cell, that is, starting idealized “base” models of OLM cells. These base models should be further explored for degeneracy and can be leveraged for additional insights and hypothesis generation moving forward (see section Methods). However, they represent the most comprehensive multi-compartment models of OLM cells to date, having been produced using morphologies and electrophysiological recordings obtained from the same biological OLM cells.

## Discussion

In this work, we obtained a set of recordings from OLM cells in hippocampal CA1 that allowed us to explicitly link morphological, passive, and h-channel biophysical parameters within the same cell. From this set, we constructed three “next generation” experimentally constrained multi-compartment models of CA1 OLM cells. The models developed here are considered “next generation” in that, unlike all previous computational models of OLM cells (Skinner and Ferguson, 2018), we have here for the first time matched morphology and electrophysiology to characterize h-channels on an individual per-cell basis and, further, to constrain two full spiking models of OLM cells. At present, it is unknown whether OLM cells express h-channels on their dendrites. Our models predict that h-channels are not confined to the soma, but rather are expressed along the dendrites of OLM cells. Our models can be used in future studies to explore the synaptic and network consequences of dendritic h-channels on OLM cells within the context of hippocampal microcircuit function. Importantly, our work shows that it is possible to robustly characterize *dendritic* ion channels by tight interactions between multi-compartment model building and somatic electrophysiological recordings.

### OLM Cells: H-Channels and Hippocampal Microcircuit Operations

The existence of h-channels, mixed cation channels that activate with hyperpolarization, has long been known since first discovered as “funny” currents in the heart (Brown et al., 1979), and in the CNS, they contribute to maintenance of the resting membrane potential, pacemaking ability, and synaptic integration (Magee, 1998; Lörincz et al., 2002; Biel et al., 2009). The contribution of h-channels in pyramidal cells to subthreshold resonance and spiking output features in hippocampus and cortex has been much studied (Santoro and Baram, 2003; Biel et al., 2009; Zemankovics et al., 2010; Narayanan and Johnston, 2012). In particular, it is known that the distribution of HCN1-containing channels increases from soma to distal dendrite and as such, have been shown to control the temporal summation of synaptic inputs from dendrites to soma (Magee, 1998; Vaidya and Johnston, 2013). However, h-channels in cerebellar Purkinje neurons are uniformly distributed in their dendrites and do not strongly affect temporal summation to the soma (Angelo et al., 2007). For interneurons, and OLM cells in particular, it is known that they express HCN channels, as seen by a large sag upon hyperpolarization (Maccaferri and McBain, 1996).

H-channels in OLM cells have been implicated in pacemaking and oscillatory activities of the hippocampus (Maccaferri and McBain, 1996; Gloveli et al., 2005), and theta (4–12 Hz) rhythms in particular (Maccaferri and Lacaille, 2003; Rotstein et al., 2005). Subsequent experimental studies found that OLM cells did not have any preferred spiking frequency response to broadband artificial synaptic inputs (Kispersky et al., 2012). Kispersky et al. (2012) did find, however, that OLM cells exhibited a phase-locked spiking preference to theta frequency modulated inputs, but this spike resonance did not depend on h-channels. However, these frequency modulated synaptic inputs were delivered exclusively to the soma of OLM cells via dynamic clamp technology. Using computational model databases of OLM cells in the absence or presence of h-channels in dendritic compartments revealed that OLM cells modeled to be in a simplified *in vivo*-like scenario could exhibit a theta frequency spiking resonance when inputs were delivered to their dendrites (Sekulić and Skinner, 2017). We further found that a high or low theta frequency spike resonance was possible and is respectively dependent on whether h-channels were present in the dendrites or not of the OLM cell models, reminiscent of Type 1 and 2 theta rhythms in the behaving animal (Kramis et al., 1975). Our modeling work examining dendritic distributions of h-channels in OLM cells found that the distributions could vary so long as total conductance was conserved (Sekulić et al., 2015), as was also found in Purkinje cells (Angelo et al., 2007). Thus a motivating factor in the present study was to constrain this extra “free parameter” by obtaining direct measurements of the total conductance in OLM cells. In doing this, we were able to show that our OLM cell models best matched the experimental data if h-channels are present in the dendrites. Interestingly, while the total h-channel conductance ranged from 2.2–4.2 nS in the three cells that were fully analyzed (Table 4), the conductance density in each of the three cells is about 0.1 pS/μm^2^, which is the value found in highly ranked OLM cell models from our previously developed model databases (Sekulić et al., 2014; Sekulić and Skinner, 2017). Zemankovics et al. (2010) obtained total conductance values averaging approximately 4 nS, which are near the upper limit of our measurements. However, total h-channel conductance values were obtained in OLM cells in rat, which had a two-fold larger measured capacitance (208 pF) than our mouse OLM cells (107 pF). Therefore, given the difference in measured surface area between rat and mouse, our data is in accordance with this previous study. Compared with Maccaferri and McBain (1996) and Zemankovics et al. (2010) who obtained, respectively, mean reversal potentials of −32.9 and −37 mV, activation curves with mean half-activation voltages (*V*_1/2_) of −84.1 and −97.7 mV, and slope factors (*k*) of −10.2 and −8.9 mV, our reversal potentials ranged from −25.2 to −34 mV, with model *V*_1/2_ fits of −99.6 to −113.8 mV, and *k* of −9.99 mV (Table 4). The voltage-dependence of the time constant yielded fits that were different but with overlapping values for the three cells (Figure 3C). A direct comparison of steady-state activation and voltage-dependent time constants in previous and present models are provided in Supplementary Figure 3C.

It has been proposed that OLM cells play a gating role (Leão et al., 2012), akin to earlier work by Blasco-Ibáñez and Freund (1995) who showed that “horizontal interneurons” (i.e., putative OLM cells) could act as a switch controlling activation of local pyramidal cells via Schaeffer collaterals or perforant path input from entorhinal cortex. Further work has shown that OLM cells in intermediate regions of CA1 exert a bidirectional control on learning and memory (Siwani et al., 2018), and ventral OLM cells control Type 2 theta rhythms and are associated with increased risk-taking (Mikulovic et al., 2018). In a recent modeling study, OLM cells were shown to be critical in producing a robust intrinsic theta output (Chatzikalymniou and Skinner, 2018), which suggests that their neuromodulation may be key to the maintenance of theta rhythms.

In building our next generation OLM cell models using morphological and electrophysiological data from the same cell, we were able to robustly show, and thus predict, the presence of h-channels in the dendrites of OLM cells. In doing this, it was critically important that the experimental data came from the same cell. OLM cells have been discovered to be comprised of parvalbumin- and 5-HT_3A_ receptor subtypes (Chittajallu et al., 2013). With the advent of sophisticated genetic sequencing techniques (Harris et al., 2018; Cembrowski and Spruston, 2019), additional OLM cell subtypes can be recognized. It has been found that CA1 pyramidal cells have a continuous, rather than discrete, variation on the longitudinal axis of the hippocampus, indicating this as an organizational principle (Cembrowski et al., 2016), and structural-functional correlations are apparent for ventral, intermediate and dorsal regions of the long axis (Fanselow and Dong, 2010). Given this observation, it is interesting to note that *Cell 1* and *Cell 2* from an intermediate CA1 region have more similar characteristics than *Cell 3* which is from a more ventral CA1 region (see Figure 2). This suggests that hippocampal CA1 interneurons may also exhibit gradients in channel, morphology, or physiological features along the hippocampal longitudinal axis, although further study is needed to verify this.

### Exposing and Exploiting Limitations in Experiments and Multi-Compartment Model Development, and a “Cycling” Strategy

It was initially unexpected that a model with fitted passive properties and morphologies obtained in conjunction with h-channel parameters extracted from the same cell did not capture corresponding experimental voltage traces (Figure 3A). To explain why this may be the case, some general issues in building multi-compartment models directly from limited experimental data need to be considered.

In an attempt to constrain as many distinct parameters within the same cell as possible, we deliberately sacrificed depth for breadth so that practical choices were inevitable in the distribution of efforts. Thus the quality of the passive and h-channel data obtained from experimentally recorded OLM cells was not optimal. For example, there are inherent limitations to cell stability that require rapid succession through a sequence of experimental protocols (Table 1). In our hands, the limit of stability was approximately 30 min. In this time, we were able to obtain recordings, bath changes, and biocytin fills that allowed us to do reconstructions, and obtain passive property and h-channel biophysical properties, but having several protocols prevented multiple sweeps of any given protocol. The I–V relation for determining maximum conductance and reversal potential was not always linear across all voltage steps, as required from theoretical perspectives in the mathematical model formulations. Furthermore, there was some error associated with fitting the Boltzmann function describing the steady-state activation curves to all data points obtained from the h-channel activation protocol. Indeed, due to inherent biological variability and experimental constraints, some measure of error is expected whenever experimental data is fitted to theoretical or mathematical models, such as a Boltzmann function for the activation curves, or a dual exponential function for the time constant of activation. Accordingly, although we obtained the requisite experimental data for fully characterizing h-channels and fitting to mathematical models of them, we should not expect that the resulting parameters will necessarily result in fully appropriate cellular output when initially used. That is, even when inserted into multi-compartment models built of the same cells from which the h-channel characteristics were obtained, there may be error in the resulting model's *V*_*m*_'s output compared to that of the experimental recordings. In essence, this is due to the accumulation of errors in estimating the various parameters used, and is compounded with increasing number of experimentally-constrained parameters in the model.

To overcome this, we found that an approach of a staggered re-fitting of the parameters in the model was able to produce generalizable results so that the *V*_*m*_ output could match all of the experimental traces including those for which it was not specifically optimized. This approach can be thought of as correcting for errors in the procedures for extracting the parameter values from the experimental data. Having many recordings from the same cell allowed us to do a staggered re-fitting of model parameters that avoided overfitting and allowed validation (see Appendix 2 in Supplementary Material), as well as consideration of the voltage dependence of h-channel activation time constants. It may be possible to use more sophisticated optimization schemes to obtain generalized fits, but the challenge of fitting detailed multi-compartment models with many parameters to experimental data is recognized, and has led to use of two-stage fitting processes (Roth and Bahl, 2009; Hay et al., 2011). We note that our staggered re-fitting can be considered as a form of two-stage fitting where in our situation, we determined how to proceed with the re-fitting stages based on how robust the experimental recordings were considered to be.

Clearly, it is important to keep in mind what one's goal(s) are in the building of a multi-compartment model in the first place. Without making some simplifying assumptions, such as uniform passive properties, and having constraining experimental data, we are necessarily faced with the curse of dimensionality (Almog and Korngreen, 2016). In our original multi-compartment models of OLM cells (Saraga et al., 2003), we were motivated to include dendrites because of clear evidence of highly active dendrites (Martina et al., 2000) in OLM cells. Moving forward, we expanded the extent of ion channels present in the models when experimental data specific to M-channels in OLM cells was available (Lawrence et al., 2006). A key notion in experimentally-constrained computational modeling is that the models are never complete. A reciprocal transfer of knowledge between model and experiment where experimental data is used to constrain models which, in turn, both point out gaps in our understanding of the underlying cellular neurophysiology as well as generate hypotheses, refine protocols, and consider additional measurable parameters that can then be incorporated into future model revisions.

A particular conceptualization of the role of computational modeling in neuroscience is to help resolve, or at least reframe, these basic concerns of how “realistic” detailed models can be. Rather than the idea of obtaining a detailed model as a crystallized end point of any given study, we consider the role of the detailed modeling as an integral component of a cyclical process of knowledge generation in neuroscience. We have expressed this as an experiment-modeling cycling approach (Sekulić and Skinner, 2018) and we consider that an essential goal in multi-compartment modeling is the back-and-forth cycling between experiments and models that leads to continual refinement of the model relative to the biological cell, thus allowing for the generation of predictions for further experiments.

### Limitations and Future Work

Although doing more than three full reconstructions, analysis and multi-compartment model building may be desirable, we felt that consistently obtaining best matches with dendritic h-channels in all three of our models when fit with data from the same cell was enough to allow for predictions as to dendritic expression of h-channels in OLM cells. Also, we focused on uniform h-channel distribution in the dendrites since our starting models using either no h-channels or h-channels fully and uniformly distributed in the dendrites did not match the experimental data (Figure 3A). Considering distributions that were not uniformly distributed (e.g., distributed only in proximal dendrites) would be unlikely to capture the data given that the total h-channel conductance would remain the same. It is also possible that decreasing or increasing conductance density distributions may be present in the dendrites, but this was not specifically explored here as additional parameters to fit would be required (see previous computational explorations in Sekulić et al., 2015), which could confound the staggered re-fitting process. Indeed, a limitation of the electrophysiological data is that our recordings were somatic. Due to the relatively compact nature of OLM cell dendrites, this is not a major limitation unlike what it may be for pyramidal cells which have extended dendritic trees. Specifically, looking at attenuation in the OLM cell models as well as previous work (Lawrence et al., 2006), space clamp is expected to be good up to at least 100 μm. Placing h-channels only in the soma failed to capture the experimental results. In the end, we obtained a strong prediction of h-channels being present in the dendrites since all three individual model fits supported this interpretation, indicating the robustness of our staggered re-fitting procedure.

Our development of full spiking OLM cell models here, as based on *Cell 1* and *Cell 2*, are available for future use. In particular, it would be interesting to use currentscape visualization analyses (Alonso and Marder, 2019) to help disentangle the interacting dynamics, perhaps using it to direct how one might best reduce the model complexity to allow dynamical system analyses to be applied, as well as applying sensitivity analysis techniques such as uncertainpy (Tennøe et al., 2018). In turn, this could help decipher how OLM cells preferentially respond to different theta frequencies based on their biophysical profile as shown in our previous computational models (Sekulić and Skinner, 2017). Further, these models now provide a foundation or canonical start for the creation of new databases designed to address specific biophysical questions as done in our original database that was developed to ask whether h-channels were present in the dendrites (Sekulić et al., 2014). Interestingly, co-regulation of h-channels and A-type channels are apparent in the currentscapes (see Figure 6B) as was observed in our original OLM cell databases.

A discrepancy between model and experimental outputs has to do with the post-inhibitory rebound responses in *Cell 2* and *Cell 3*. These rebound responses are unlikely to be solely due to *I*_*h*_ as the models with only *I*_*h*_ do not capture this aspect in *Cell 2* or *Cell 3* for example (see Figure 3C). This indicates that other currents such as T-type calcium currents that were not blocked (see Table 1) are contributing to rebound responses in *Cell 2* and *Cell 3*, but less so for *Cell 1*. Given that calcium currents, calcium-activated potassium currents and calcium handling aspects in previous OLM cell models did not have any direct experimental data constraints (Lawrence et al., 2006), we elected not to focus on calcium specifics here, but rather on the spiking activities with the existing current models and adjusting only the maximal conductance values, as described in detail in the Methods. Preliminary simulations with *Cell 2* indicates that increases in T-type calcium conductances on its own can produce rebound spikes, but this necessarily makes other spiking features inappropriate. For the models to more fully capture electrophysiological responses, one could consider using these models to build new model populations, but now with calcium currents rather than *I*_*h*_ as the focused question (Sekulić and Skinner, 2018). It would also be helpful to acquire data on axonal properties of OLM cells beyond Martina et al. (2000) to figure out their properties, and new theoretical insights could perhaps be harnessed (Goethals and Brette, 2020).

We have previously shown that using virtual networks, or creating *in vivo*-like representations with multi-compartment cellular models, as done with our earlier OLM cell models (Sekulić and Skinner, 2017), can lead to insights of circuit function from cellular specifics. Further, we have now used the current OLM cell models to do detailed, dendritic explorations of ion channel dynamics that would not be possible to do experimentally (Guet-McCreight and Skinner, 2020). We have also created *in vivo*-like states with interneuron-specific interneuron models (Guet-McCreight and Skinner, 2019), and used them to make links between *in vitro* and *in vivo* studies (Luo et al., 2020). In essence, it seems possible that an understanding of the contribution of biophysical cellular details to circuits in the behaving animal can emerge by using virtual networks.

In a review, Almog and Korngreen (2016) demonstrate the limitations associated with the re-usability of layer 5 pyramidal cell models, and also state that there is a need for proving that multi-compartment models are valid within the context of network simulations. These are challenging issues to consider but an important step that they suggest is to ensure that models are linked with the experimental data. Along these lines, neuroinformatic tool developments (e.g., Nexus—https://bluebrainnexus.io) can help reduce the workload.

In conclusion, our work has shown that if the development of multi-compartment models are done for a specific cell type in which ion channel characterization and morphological and passive data can be obtained from the same cell, it is possible to determine their ion channel distribution and biophysical characterization from somatic recordings alone. In this way, one can envisage doing this in a cyclic fashion to characterize other ion channel types and distributions that are unknown.

## Data Availability Statement

The datasets presented in this study can be found in online repositories. The names of the repository/repositories and accession number(s) can be found below: NEURON code for all the models are available at: https://github.com/FKSkinnerLab/OLMng and associated experimental data available at: https://osf.io/qvnu9/.

## Ethics Statement

The animal study was reviewed and approved by University of Montana, Texas Tech University.

## Author Contributions

FS: conceptualization, resources, supervision, funding acquisition, validation, writing–original draft, project administration, writing–review, and editing. VS: conceptualization, resources, data curation, software, formal analysis, validation, investigation, visualization, methodology, writing–original draft, project administration, writing–review, and editing. JL: conceptualization, resources, supervision, funding acquisition, writing–original draft, project administration, writing–review, and editing. AG-M: conceptualization, software, formal analysis, investigation, visualization, methodology, writing–original draft, writing–review, and editing. FY and TG: conceptualization, data curation, formal analysis, investigation, writing–review, and editing. All authors contributed to the article and approved the submitted version.

## Conflict of Interest

The authors declare that the research was conducted in the absence of any commercial or financial relationships that could be construed as a potential conflict of interest.
