# Supplementary material for: Integration of Within-Cell Experimental Data With Multi-Compartmental Modeling Predicts H-Channel Densities and Distributions in Hippocampal OLM Cells
Source: Front Cell Neurosci. 2020 Sep 17;14:277. doi: 10.3389/fncel.2020.00277 (PMC7527636; doi:10.3389/fncel.2020.00277)
Supplement: Supplementary file 1 [file Data_Sheet_1.PDF]

Supplementary Material

APPENDIX 1

Scaling dendrites to consider low specific conductance estimates

From our model fits we found that the specific conductances (C_m's) obtained were lower than the $\approx$ 0.9-1μF/cm^2 that have been previously reported as a "standard" value in mammalian neurons (Gentet et al., 2000). Since a particularly prominent source of possible errors in morphological reconstruction of neurons is in dendritic diameters (Jaeger, 2001), we performed a simple scaling exercise on dendritic diameters. For instance, although fine dendritic processes with diameters between 0.5 and 2μm can be resolved using confocal microscopy, their apparent diameters will generally seem larger than their true size due to the point spread function of the optical system (Jacobs et al., 2010). To assess whether our low fitted C_m values reflected compensation for overestimated dendritic diameters or whether they may reflect lower specific capacitance in biological OLM cells, we turned to cable theoretic considerations.

Holmes et al. (Holmes et al., 2006) showed that when either the diameter d or length l are multiplied by a constant factor x, the derived changes in membrane resistivity (R_m), axial resistivity (R_a), and C_m needed to maintain an identical voltage response occur when R_m is scaled by x, C_m by $1/x$, and R_a by x^2. Note that since leak conductance (G_{pas}) corresponds to the inverse of R_m, the necessary change for G_{pas} is rather to scale it by $1/x$. If we consider re-scaled dendritic diameters of our OLM models by scaling values (d_s) of 0.5 and 1.5 to represent large changes, and 0.9 and 1.1 to represent small changes, the resulting values of G_{pas}, C_m, and R_a as predicted by cable theory are shown in Table S1. To confirm these predictions, we first re-scaled the original reconstructed diameters for each compartment in two of the models (*Cell 1* and *Cell 2*) using these four scaling values (0.5, 1.5, 0.9, 1.1). We used our unchanged passive properties previously obtained and examined the responses to a -120pA current clamp step. This served to assess the resulting changes in the V_m responses attributable to errors in electrotonic properties solely due to changing the diameters. We found that changes in diameters produced deviations in the V_m response roughly proportional to the magnitude of the scaled dendritic diameters (Fig S1A). The largest change was when the diameters were halved across all compartments (d_s = 0.5). We then re-fitted the passive properties of the two models under each case of re-scaled dendritic diameters, obtaining nearly identical model V_m responses to the -120pA current clamp step (Fig S1B). We found that the refitted values of R_m, C_m, and R_a for the various cases of scaled dendritic diameters, d_s, were in excellent agreement with the values predicted by cable theory (Table S1). Thus, we were able to use the cable theoretic predictions to provide implicit limits on how much the fitted parameters could be expected to deviate solely due to errors in morphological considerations.

All of our models were found to have consistent but low C_m values as given in the main text (0.27 for *Cell 1*, 0.28 for *Cell 2*, and 0.31 for *Cell 3*, units of μF/cm^2). Let us suppose that the diameter estimates in our morphological reconstructions were nevertheless overestimates of the true diameters. Then, the cable theoretic predictions, as confirmed by our simulations, would suggest that even if we halved the average diameters, the expected C_m in the cells would only increase to about 0.58 μF/cm^2, taking the average of the re-fitted C_m of *Cell 1* and *Cell 2*. It is unlikely that our diameters were this much in error. That is, a scaling factor of 0.5 implies that the minimum diameter, which is 0.35 μm in *Cell 1* and 0.32 μm in *Cell 2*, would had to have been reduced to 0.17 μm and 0.16 μm for *Cell 1* and *Cell 2*, respectively,

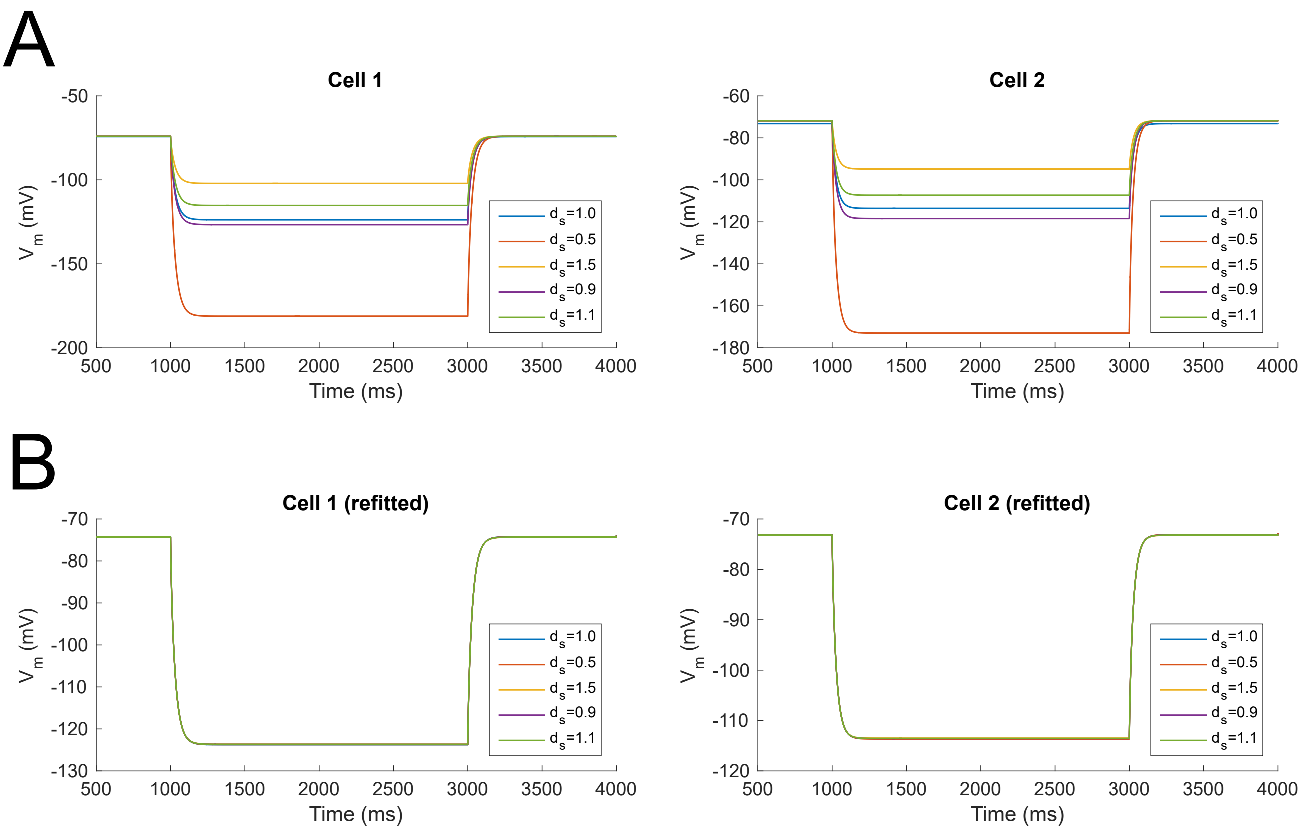

Figure S1. Model membrane potential responses with scaled dendritic diameters and both unchanged and refitted passive properties. Responses of two models (*Cell 1*, left and *Cell 2*, right) to -120pA current clamp step commands and a wide range of rescaled dendritic diameters and with **A.** original (fixed) and **B.** refitted passive properties. The scaling factor, d_s, represents the constant scaling factor performed on each compartment's diameter in the respective cell reconstruction. The response of the original reconstructed model – i.e., with no scaling – is shown as d_s=1.0 for all cases.

Table S1. Re-fitted passive properties for models with scaled dendritic diameters.

		fitted values					***cable theory***		
	d_s	*RMSE* (mV)	R_a (Ωcm)	C_m (μF/cm^2)	G_{pas} (S/cm^2)	E_{pas} (mV)	R_a* (Ωcm)	C_m* (μF/cm^2)	G_{pas}* (S/cm^2)
Cell 1	***1.0***	*0.3602*	*141.85*	*0.2698*	*7.933×10^{-6}*	*49.05*			
	0.5	0.3665	36.87	0.5278	1.556×10^{-5}	49.09	35.46	0.5396	1.586×10^{-5}
	1.5	0.3959	386.51	0.1592	4.768×10^{-6}	49.28	319.16	0.1799	5.288×10^{-6}
	0.9	0.3766	121.35	0.2802	8.342×10^{-6}	49.18	114.89	0.2998	8.814×10^{-6}
	1.1	0.3827	195.82	0.2269	6.750×10^{-6}	49.20	171.63	0.2453	7.211×10^{-6}
Cell 2	***1.0***	*0.9818*	*285.78*	*0.2799*	*9.242×10^{-6}*	*54.74*			
	0.5	0.9104	64.78	0.6484	2.085×10^{-5}	54.60	72.44	0.5598	1.848×10^{-5}
	1.5	1.0486	707.21	0.1624	5.480×10^{-6}	54.85	643.01	0.1866	6.161×10^{-6}
	0.9	0.9676	226.97	0.3201	1.051×10^{-5}	54.71	231.48	0.3110	1.029×10^{-5}
	1.1	0.9958	350.50	0.2470	8.198×10^{-6}	54.77	345.79	0.2545	8.401×10^{-6}

d_s is the scaled diameter. R_a, C_m* and G_{pas}* are the values as predicted from cable theory and are given by $R_a* = R_a \times d_s^2$, $C_m* = C_m \times 1/d_s$, and $G_{pas}* = G_{pas} \times 1/d_s$*

which are unreasonably small. If we nevertheless consider these scaled diameter values as possible ranges, we are led to the consideration that C_m in OLM cells may be within the range of 0.2-0.6 μF/cm^2. We further note that if we compute C_m for these three cells directly from experimental capacitance and surface area values, then they are also low, as shown in Table S2. We thus decided to keep the values of C_m obtained from the passive property fitting procedure for each model and built upon this passive backbone. It may be worthwhile to experimentally explore these low capacitance results in the form of nucleated patch recordings as done previously for directly measuring the specific capacitance in other neurons (Eyal et al., 2016; Gentet et al., 2000). It is interesting to note that in the case of human neurons where values of $\approx$0.5 μF/cm^2 were reported (Eyal et al., 2016), another group has reported values of $\approx$0.9 μF/cm^2 (Beaulieu-Laroche et al., 2018).

Table S2. Specific capacitance computed directly from experiment.

Parameter	***Cell 1***	***Cell 2***	***Cell 3***
Capacitance (pF)	62.8	123.7	79.6
Surface area (μm^2)	29,378.1	35,158.5	21,990.3
C_m (μF/cm^2)	0.21	0.35	0.36

Capacitance properties were extracted at a current injection of -60 pA.

REFERENCES

Beaulieu-Laroche, L., Toloza, E. H. S., van der Goes, M.-S., Lafourcade, M., Barnagian, D., Williams, Z. M., et al. (2018). Enhanced Dendritic Compartmentalization in Human Cortical Neurons. *Cell* 175, 643–651.e14. doi:10.1016/j.cell.2018.08.045

Eyal, G., Verhoog, M. B., Testa-Silva, G., Deitcher, Y., Lodder, J. C., Benavides-Piccione, R., et al. (2016). Unique membrane properties and enhanced signal processing in human neocortical neurons. *eLife* 5, e16553. doi:10.7554/eLife.16553

Gentet, L. J., Stuart, G. J., and Clements, J. D. (2000). Direct Measurement of Specific Membrane Capacitance in Neurons. *Biophysical Journal* 79, 314–320. doi:10.1016/S0006-3495(00)76293-X

Holmes, W. R., Ambros-Ingerson, J., and Grover, L. M. (2006). Fitting experimental data to models that use morphological data from public databases. *Journal of Computational Neuroscience* 20, 349–365. doi:10.1007/s10827-006-7189-8

Jacobs, G., Claiborne, B., and Harris, K. (2010). Reconstruction of Neuronal Morphology. In *Computational Modeling Methods for Neuroscientists*, ed. E. De Schutter (Cambridge, MA: MIT Press). 187–210

Jaeger, D. (2001). Accurate reconstruction of neuronal morphology. In *Computational Neuroscience: Realistic Modeling for Experimentalists*, ed. E. De Schutter (Boca Raton, Fla: CRC Press). 159–178
