# Supplementary material for: Integration of Within-Cell Experimental Data With Multi-Compartmental Modeling Predicts H-Channel Densities and Distributions in Hippocampal OLM Cells
Source: Front Cell Neurosci. 2020 Sep 17;14:277. doi: 10.3389/fncel.2020.00277 (PMC7527636; doi:10.3389/fncel.2020.00277)
Supplement: Supplementary file 2 [file Data_Sheet_2.PDF]

Supplementary Material

APPENDIX 2

Overfitting and staggered re-fitting considerations and illustrations

The approach of inappropriately attributing errors to parameters, taken to its extreme, would be to allow all parameters to be adjusted simultaneously. We demonstrate the results of this "naïve" scheme by allowing the passive properties (4 parameters) and I_h properties (9 parameters) to simultaneously vary while using the PRAXIS fitting procedure in NEURON to minimize the error in V_m response between model and experiment. Let us use *Cell 3* as an example. When first fitted to the -120pA current clamp step TTX trace, the model exhibited a remarkably good fit to the experimental V_m trace (see Fig S1A, left) which, at first glance, would indicate that the OLM cell's output had been captured. However, when we then injected a -90pA current clamp step in the model with these fitted parameters as a test and compared its output to that of the experimental cell, we saw that it was very poor at matching the -90pA TTX current trace response (Fig S1A, right). If we instead fitted to the -90pA current clamp TTX trace, we found that the model could also match the experimental V_m output quite well (Fig S1B, right) but then it failed to capture the -120pA output when used as a test (Fig S1B, left). It is important to note that in doing the fits and tests, we ensured that the holding current applied to the model was always in line with what was used for the particular cell at the given current step, although fitted parameters were not changed between fits and tests. This "overfitting" of the experimental data used for adjusting the parameters was thus inappropriate, and a more judicious procedure was required, where only a subset of parameters were considered at any given time and for any given feature of the V_m mismatch between model and experiment.

In consideration of a re-fitting procedure, we noted that if we wanted to judiciously tune individual parameters in such a manner that for a given portion of each V_m trace, the parameters that can be responsible for affecting that portion of the trace should be tuned in the order from those with the greatest uncertainty to those with the least uncertainty. Using as an example the (passive property) scenario of the initial charging portion of the membrane upon step hyperpolarizing current, although we would expect all of C_m, R_a, τ_h and r_∞ to affect this portion of the trace to various degrees, it would have been a mistake to fit, say, r_∞ prior to fitting C_m. This is not only because I_h wouldn't yet be fully activated but also because the fitted function for r_∞ exhibits a very good match to the recorded steady state current values for multiple voltage steps (See Fig 3C in main paper text), whereas the fit for C_m has more uncertainty due to the issues of dendritic diameter estimation as well as cell rundown seen in the recordings used for fitting, as previously described. If we were to have fitted r_∞ first, we would have attributed an undue source of error of the mismatch in V_m to r_∞. The disproportionate change in r_∞ curve would then have manifested in inappropriate V_m output elsewhere. Therefore, the criterion for whether we had selected an appropriate parameter for re-fitting was that if the re-fitted parameter resulted in a better fit to the portion of V_m trace under consideration but a worse fit elsewhere, then we had not selected the correct parameter for which the error in V_m mismatch should be attributed to. In practice we could perform this test by fitting to one set of current clamp traces and validating the model's correctness by testing its output to another current clamp step trace without re-fitting the parameter. Our staggered re-fitting and test validation for the three model cells are shown in Fig S2, Fig S3, and Fig S4.

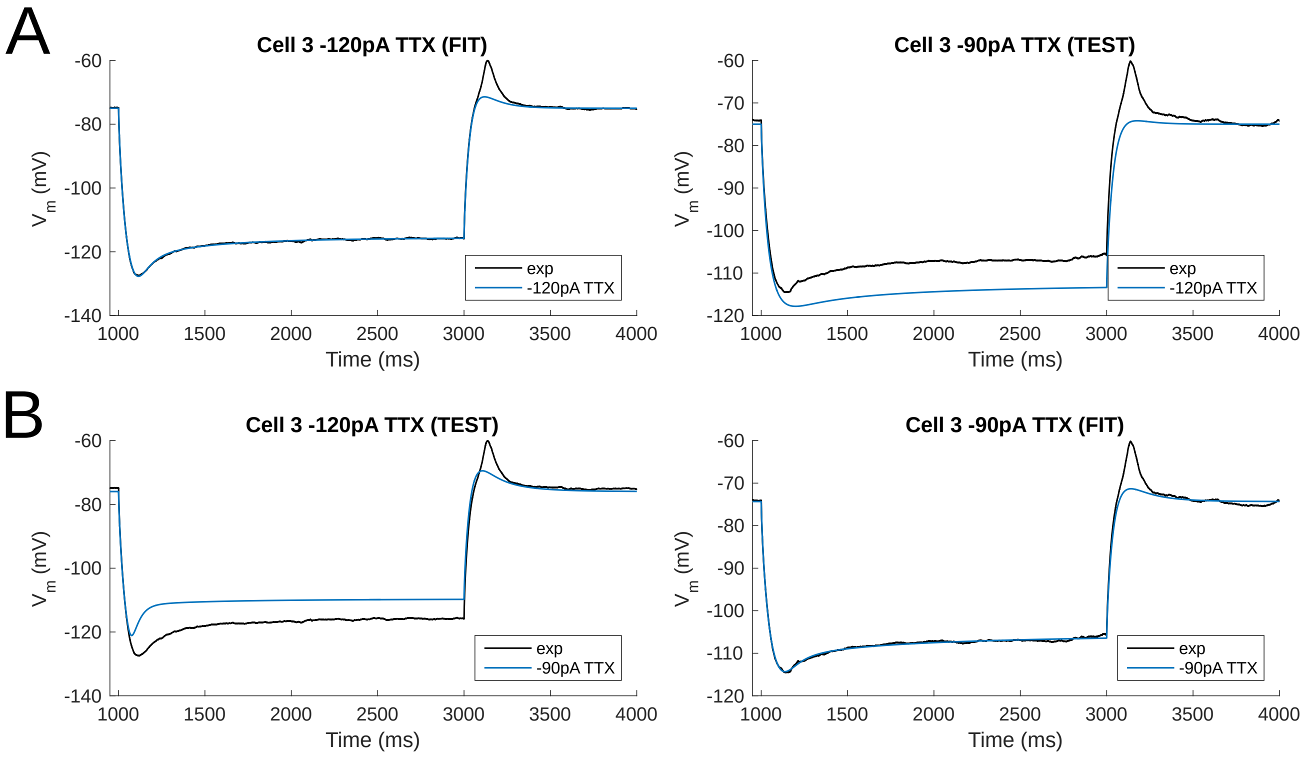

Figure S1. Simultaneous fitting of all passive and I_h parameters leads to overfitting of the experimental traces and poor model generalization - Cell 3 as an example. A: Model fitted to a -120pA TTX current clamp trace (left) and tested against a -90pA current clamp trace (right). B: The reverse case, with model fitted to a -90pA TTX current clamp trace (right) but tested against a -120pA trace (left). H_{dist}=1. Holding current injections: 2.7 pA for -120pA step; 3.1 pA for -90pA step.

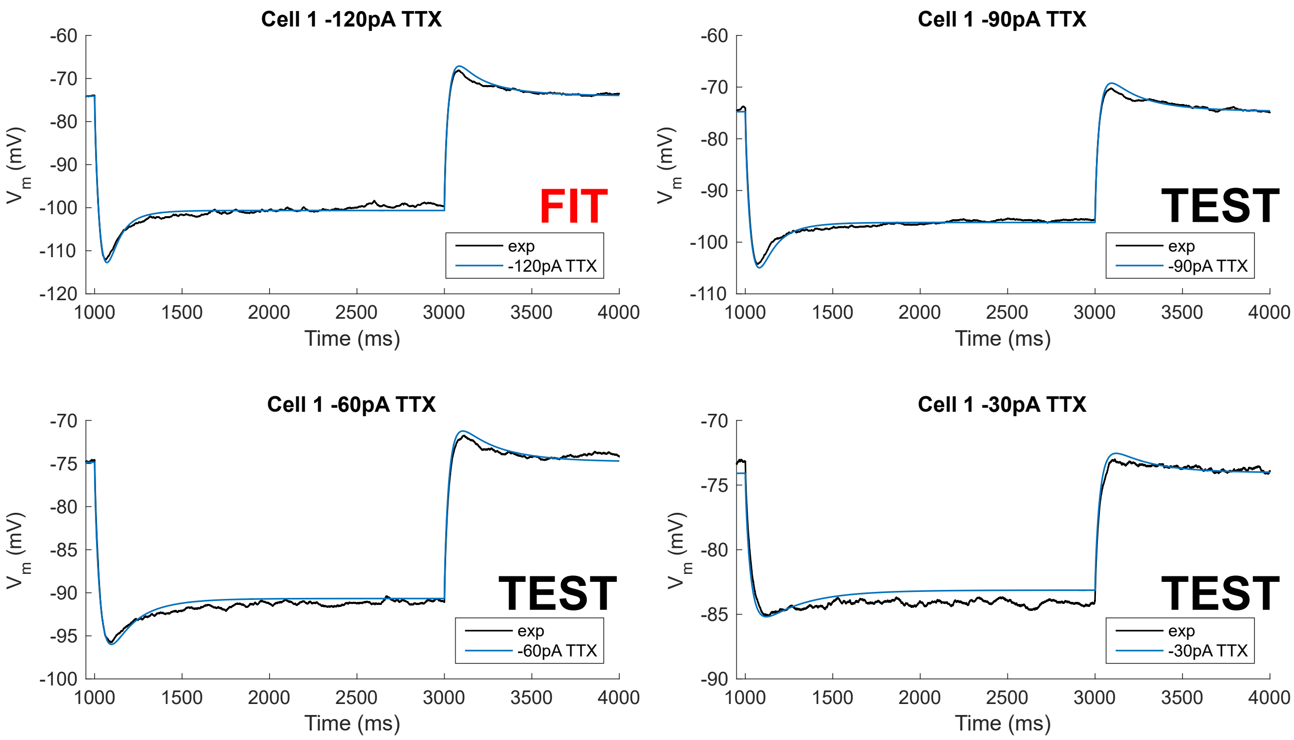

Figure S2. Traces for Cell 1 model compared to experiment after staggered re-fitting. Model V_m traces compared to experiment for *Cell 1* with staggered re-fitting procedure, where first passive properties are fitted, followed by total G_h, r_∞ and τ_h. Only the -120pA TTX trace was used for fitting; the other traces show validation of the model's parameters using different current clamp steps. H_{dist}=1. Holding current injections: -28 pA for all four steps of -120, -90, -60, -30 pA.

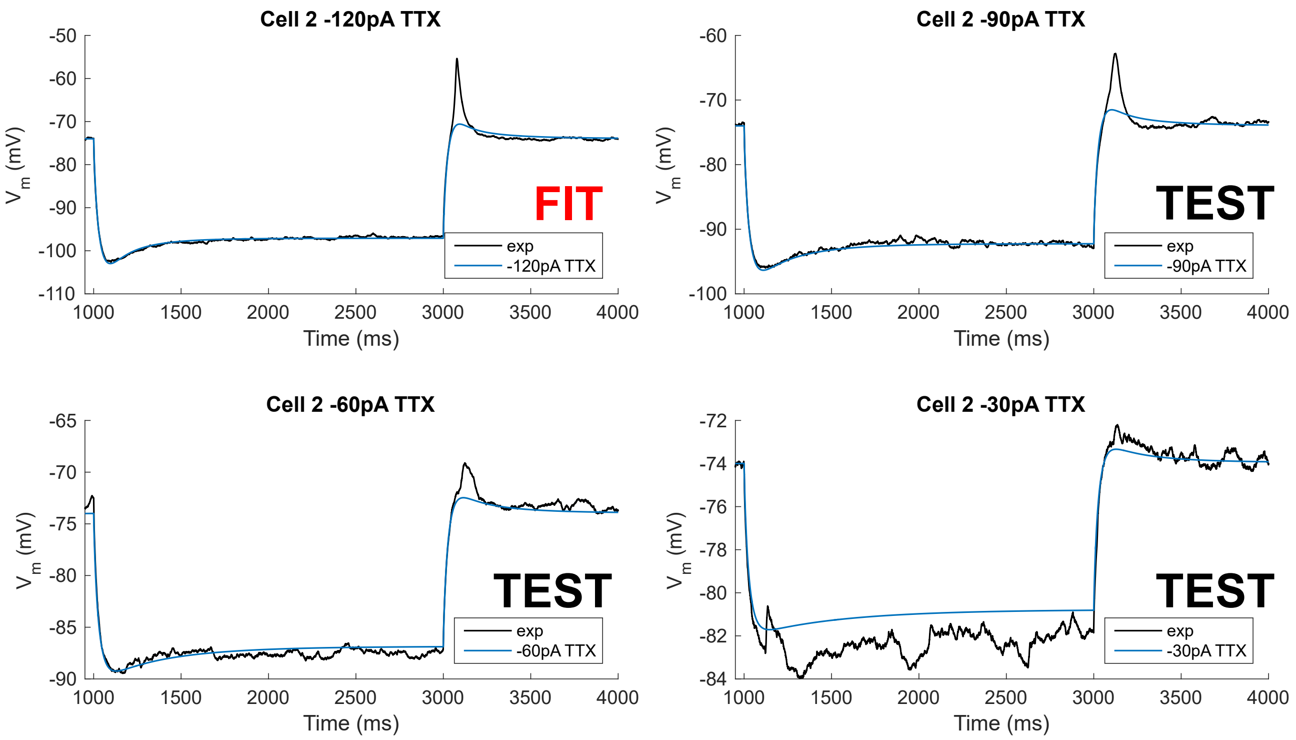

Figure S3. Traces for Cell 2 model compared to experiment after staggered re-fitting. Model V_m traces compared to experiment for *Cell 2* with staggered re-fitting procedure, where first passive properties are fitted, followed by total G_h, r_∞ and τ_h. Only the -120pA TTX trace was used for fitting; the other traces show validation of the model's parameters using different current clamp steps. H_{dist}=1. Holding current injections: -5.1 pA for all four steps of -120, -90, -60, -30 pA.

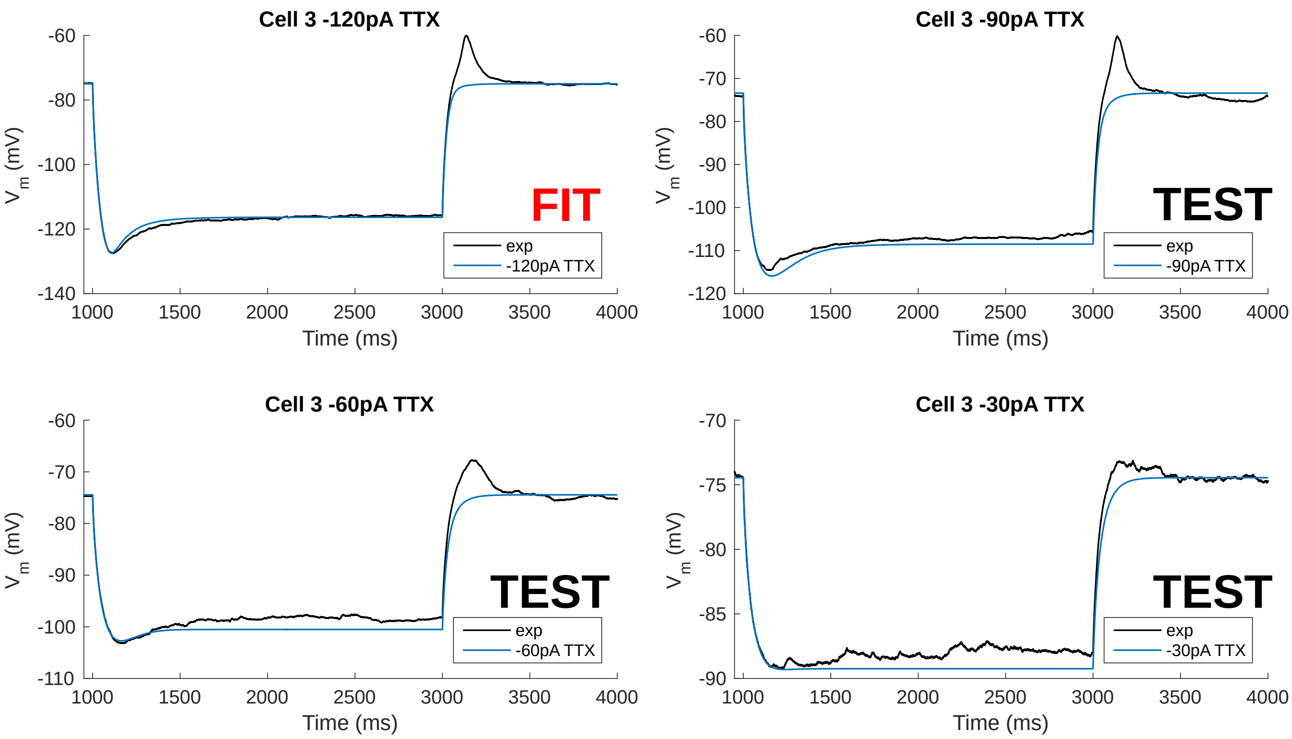

Figure S4. Traces for Cell 3 model compared to experiment after staggered re-fitting. Model V_m traces compared to experiment for *Cell 3* with staggered re-fitting procedure, where first passive properties are fitted, followed by total G_h, r_∞ and τ_h. Only the -120pA TTX trace was used for fitting; the other traces show validation of the model's parameters using different current clamp steps. H_{dist}=1. Holding current injections: 2.7 pA for -120pA step; 3.1 pA for -90 and -60pA steps; 3.4 pA for -30pA step.
