# Supplementary material for: Integration of Within-Cell Experimental Data With Multi-Compartmental Modeling Predicts H-Channel Densities and Distributions in Hippocampal OLM Cells
Source: Front Cell Neurosci. 2020 Sep 17;14:277. doi: 10.3389/fncel.2020.00277 (PMC7527636; doi:10.3389/fncel.2020.00277)
Supplement: Supplementary file 3 [file Data_Sheet_3.PDF]

Supplementary Material

SUPPLEMENTARY TABLES AND FIGURES

Tables

Table S1. Fitted passive parameters and resulting goodness of fit of models to various current clamp step traces.

	Cell 1	*Cell 1*	*Cell 2*	*Cell 2*	*Cell 3*	*Cell 3*
"ZD trace" used for fitting	-120 pA	-30 pA	-120 pA	-30 pA	-120 pA	-30 pA
R_a (Ωcm)	141.85	258.21	285.78	474.78	94.83	329.99
C_m (μF/cm^2)	0.2698	0.2552	0.2799	0.4721	0.3057	0.4744
G_{pas} (S/cm^2)	7.93×10^{-6}	9.62×10^{-6}	9.24×10^{-6}	9.39×10^{-6}	7.48×10^{-6}	7.27×10^{-6}
E_{pas} (mV)	-49.0	-48.3	-54.7	-56.6	-69.1	-68.1
RMSE (-30 pA ZD)	0.5058	0.3289	0.6594	0.1893	1.079	0.0456
RMSE (-60 pA ZD)	7.458	5.742	0.9828	0.7092	2.410	1.398
RMSE (-90 pA ZD)	1.945	3.393	0.6155	3.829	2.194	16.89
RMSE (-120 pA ZD)	0.3602	3.440	0.9818	9.875	0.5199	50.38
RMSE (-30 pA TTX)	0.6596	0.2048	0.3108	0.6234	1.679	9.021
Cumul. RMSE (mV)	10.92	13.10	3.550	15.22	7.881	77.73

Table S2. Location and optimization ranges for ion channel types.

Conductance type	***Distribution location***	***Cell 1 range*** (pS/μm^2)	***Cell 2 range*** (pS/μm^2)
$G_{NaT,s}$	soma	10-100	10-100
$G_{NaT,d}$	dendrites	40-200	40-200
$G_{NaT,a}$	axon	40-200	40-200
$G_{Kdrf,s}$	soma	3-200	50-200
$G_{Kdrf,d}$	dendrites	3-200	50-200
$G_{Kdrf,a}$	axon	3-200	50-200
$G_{Kdrs,s}$	soma	0-0.01	0-0.01
$G_{Kdrs,d}$	dendrites	0-0.01	0-0.01
$G_{Kdrs,a}$	axon	0-0.01	0-0.01
G_{KA}	soma, dendrites	1.25-120	1.25-120
G_M	soma, dendrites	0.05-1.5	0.05-1.5
G_{CaT}	dendrites	0.625-5	0.625-5
G_{CaL}	dendrites	6.25-50	6.25-50
G_{KCa}	dendrites	1.375-11	1.375-11

Table S3. Descriptions of the eFEL measurements and the chosen standard deviation values (σ) that were used as objective features and weights in the spiking model optimizations.

Name	σ	*Description*
**1.** AP_amplitude_from_voltagebase (mV)	0.1	The height of the AP measured from voltage base.
**2.** AP_width (ms)	0.01	Width of each peak at the value of threshold.
**3.** AP_amplitude (mV)	0.1	The relative height of the AP between the peak voltage and the voltage where the first derivative is higher than 12 V/s for at least 5 points.
**4.** AHP_time_from_peak (ms)	0.1	Time between AP peaks and AHP depths.
**5.** time_to_first_spike (ms)	1	Time from the start of the stimulus to the maximum of the first peak.
**6.** voltage_base (mV)	0.1	The resting membrane potential before the current step.
**7.** AP_amplitude_change	0.001	Difference of the amplitudes of the second and the first AP divided by the amplitude of the first AP.
**8.** AP_duration_half_width (ms)	0.01	Full width at half maximum of each action potential.
**9.** AHP_depth (mV)	0.1	Relative voltage difference between the minimum AHP voltage and the voltage base.
**10.** mean_frequency (Hz)	0.1	The mean frequency of the firing rate.
**11.** AHP_slow_time	0.001	Time difference between absolute voltage values at the first after-hyperpolarization starting 5 ms after the peak and the peak, divided by interspike interval.
**12.** adaptation_index	0.001	Normalized average difference of two consecutive ISIs.

AP: Action Potential; AHP: After-Spike Hyperpolarization. Consult the eFEL manual for more details on these measurements: `https://media.readthedocs.org/pdf/efel/latest/efel.pdf`

Figures

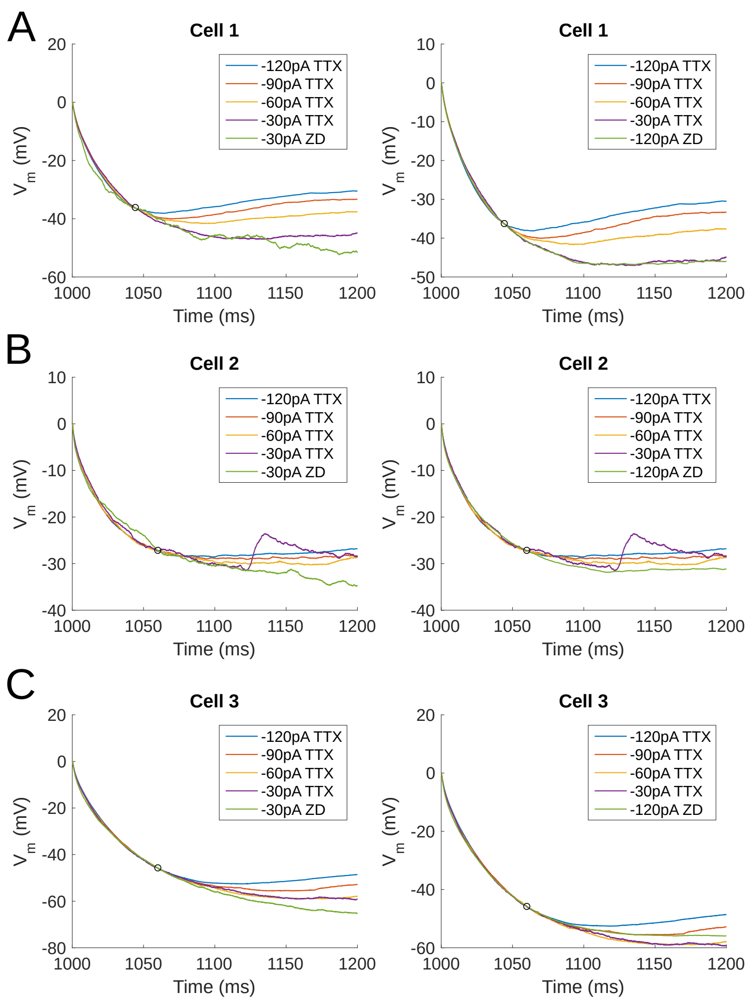

Figure S1. Comparison of charging portions of membrane potential (V_m) for fitting model passive responses. *Left column*: Traces for all hyperpolarizing current injection steps with synaptic and voltage-gated channel blockers, except ZD7288 (i.e., TTX traces as referred to in main manuscript), and the -30 pA trace with ZD7288 application (i.e., ZD traces as referred to in main manuscript) for *Cell 1, Cell 2, Cell 3* in **A, B, C**. Small circles represent the time point at which all traces were normalized and were determined by eye as the point at which depolarization due to activated h-channels caused TTX traces to deviate from the "passive" ZD condition. This value is unique per cell and relative to the time of step current injection (1000ms) as follows: 44 ms (*Cell 1*), 60 ms (*Cell 2*), 60 ms (*Cell 3*). *Right column*: As for left column, except the -120 pA ZD trace is shown instead of the -30 pA ZD trace.

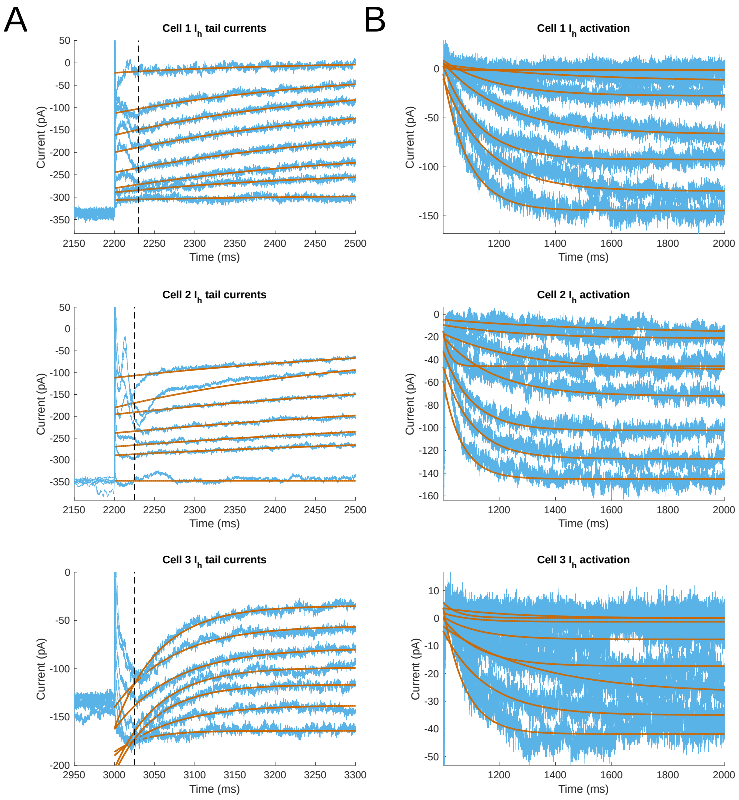

Figure S2. Experimental traces and fits for reversal potential and time constants of activation and deactivation. A: Tail current fits used for deactivation time points. **B**: I_h activation fits. Tail current protocol with leak subtraction, and ZD-subtracted traces from a voltage-clamp hyperpolarizing step protocol to reveal kinetics of I_h activation (B), for *Cell 1* (top), *Cell 2* (middle), and *Cell 3* (bottom). Fitted single exponential functions for each trace are shown in the tail current plots (A). The vertical dashed lines denote the approximate point of termination of the capacitive transient, and hence maximum deflection of the I_h current after the relaxation step in the voltage clamp protocol.

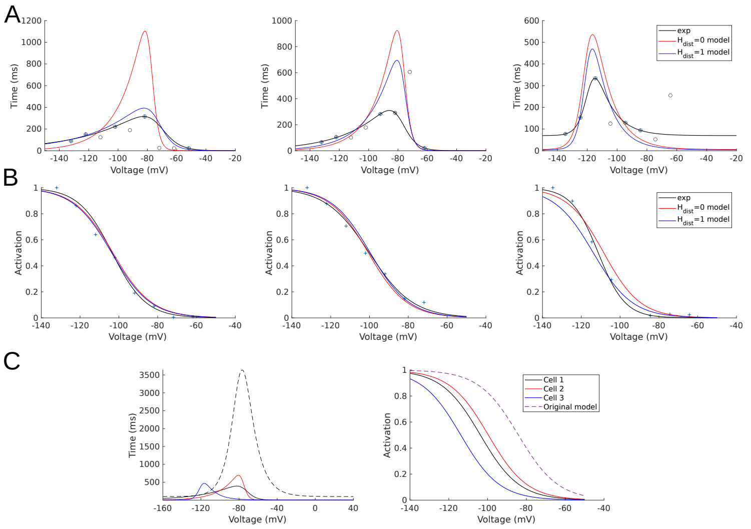

Figure S3. Comparison of h-channel parameters between models and experimental data. A: I_h time constants of activation and deactivation (τ_h) fitted with somatic or somatodendritic I_h together with all experimental data points. **B**: I_h steady-state activation curves (r_∞) fitted with somatic or somatodendritic I_h. *Cell 1, Cell 2, Cell 3* shown from left to right in (A) and (B). **C**: Model τ_h and r_∞ in final models of *Cell 1, Cell 2, Cell3* (i.e., somatodendritic I_h) of the present paper, and in OLM cell models of previous papers - *Sekulic et al. PLoS One 2014* for example.

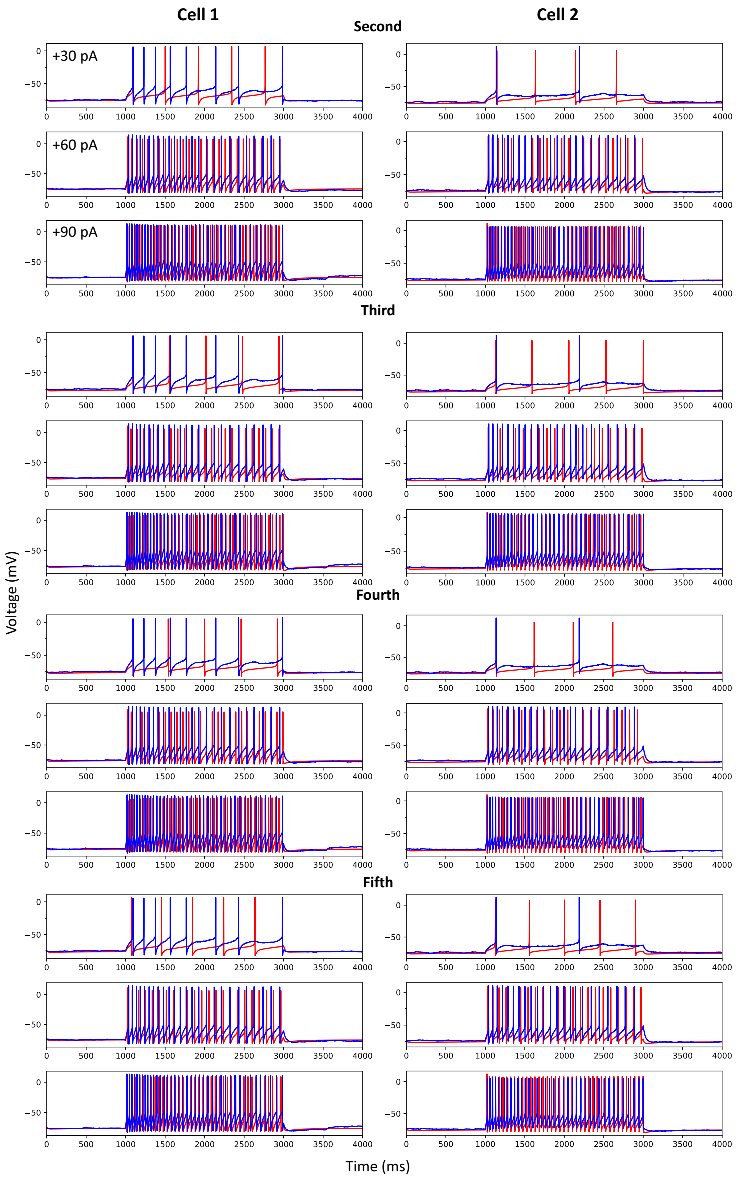

Figure S4. Other top optimized spiking models. As in main text Figure, we show the +30 pA, +60 pA, and +90 pA current injection steps for models (red) plotted against the corresponding experimental data (blue). Spiking models for *Cell 1* and *Cell 2* that were ranked second, third, fourth, and fifth are shown from top to bottom.

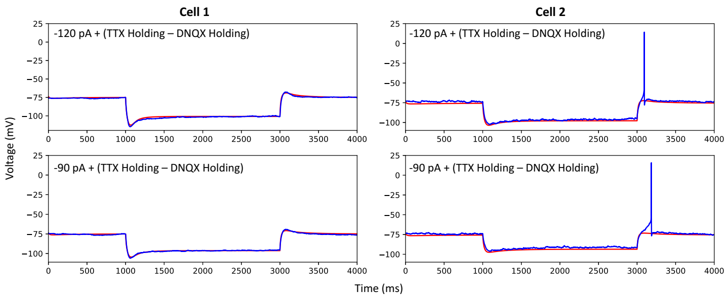

Figure S5. Adding spiking currents does not affect the fit to hyperpolarizing steps. When injecting -90 pA and -120 pA current injections to the top spiking models, we accounted for differences in holding currents during experiment with TTX traces or traces from protocol #2 in main text Table - 'DNQX current traces'. The spiking models still generate appropriate hyperpolarization responses. *Cell 1* holding current injections: -28 pA for -90 and -120 pA steps, TTX traces, and 4 pA for DNQX traces; *Cell 2* holding current injections: -5.1 for -90 and -120 pA steps, TTX traces, and -5 pA for DNQX traces.
